# Genome and transcriptome mechanisms driving cephalopod evolution

**DOI:** 10.1038/s41467-022-29748-w

**Published:** 2022-05-04

**Authors:** Caroline B. Albertin, Sofia Medina-Ruiz, Therese Mitros, Hannah Schmidbaur, Gustavo Sanchez, Z. Yan Wang, Jane Grimwood, Joshua J. C. Rosenthal, Clifton W. Ragsdale, Oleg Simakov, Daniel S. Rokhsar

**Affiliations:** 1 The Eugene Bell Center for Regenerative Biology and Tissue Engineering, Marine Biological Laboratory, Woods Hole, MA USA; 2 Department of Molecular and Cell Biology, University of California, Berkeley, CA USA; 3 Department of Neuroscience and Developmental Biology, University of Vienna, Vienna, Austria; 4 Graduate School of Integrated Sciences for Life, Hiroshima University, Higashi Hiroshima, Hiroshima, Japan; 5 Department of Neurobiology, University of Chicago, Chicago, IL USA; 6 Hudson Alpha Institute of Biotechnology, Huntsville, AL USA; 7 Molecular Genetics Unit, Okinawa Institute for Science and Technology, Okinawa, Japan; 8 Chan-Zuckerberg BioHub, San Francisco, CA USA

**Keywords:** Genome evolution, Comparative genomics, Genome

## Abstract

Cephalopods are known for their large nervous systems, complex behaviors and morphological innovations. To investigate the genomic underpinnings of these features, we assembled the chromosomes of the Boston market squid, *Doryteuthis (Loligo) pealeii,* and the California two-spot octopus, *Octopus bimaculoides*, and compared them with those of the Hawaiian bobtail squid, *Euprymna scolopes*. The genomes of the soft-bodied (coleoid) cephalopods are highly rearranged relative to other extant molluscs, indicating an intense, early burst of genome restructuring. The coleoid genomes feature multi-megabase, tandem arrays of genes associated with brain development and cephalopod-specific innovations. We find that a known coleoid hallmark, extensive A-to-I mRNA editing, displays two fundamentally distinct patterns: one exclusive to the nervous system and concentrated in genic sequences, the other widespread and directed toward repetitive elements. We conclude that coleoid novelty is mediated in part by substantial genome reorganization, gene family expansion, and tissue-dependent mRNA editing.

## Abstract (web summary)

“Cephalopods are known for their large nervous systems, complex behaviors, and morphological innovations. Here, the authors find that soft-bodied cephalopod genomes are more rearranged than other extant molluscs and that mRNA editing patterns are associated with the nervous system and repetitive elements”.

## Introduction

The complex behavioral repertoire of coleoid cephalopods (squid, cuttlefish, and octopus) is orchestrated by the largest of invertebrate nervous systems, which arose by an independent, radically different, and largely unknown evolutionary trajectory compared with that of vertebrates^1,2^. At a genomic level, vertebrate complexity has been hypothesized to be linked to repeated rounds of whole genome duplication^3,4^, but this mechanism is not in play in cephalopods^5^. Nevertheless, coleoid cephalopod chromosome numbers are dramatically larger than those of other molluscs^6^, suggesting a possible role for chromosome-disrupting processes in coleoid evolution. At the transcriptional level, messenger RNA editing has been proposed as a potent mechanism for expanding protein diversity in coleoid cephalopods^5,7–10^. In vertebrates, editing is largely limited to transcribed transposable elements; only a handful of important nervous system proteins are functionally altered by edits^11,12^. Despite differences in genome duplication and RNA editing, notable convergent gene family expansions have occurred in vertebrates and cephalopods, but using distinct mechanisms^5^. The relative contribution of these and other factors to complexity and novelty in cephalopods has remained mysterious, in part due to the lack of complete chromosome-scale genome sequences and an absence of sampling of RNAs across tissues.

**Fig. 1:**
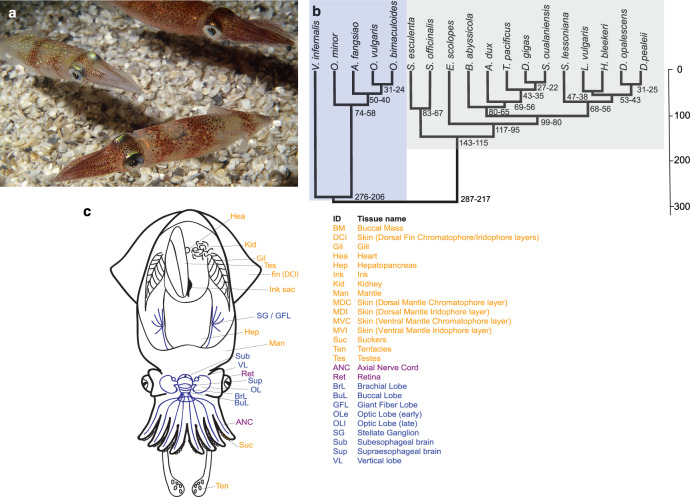
*Doryteuthis pealeii* anatomy and phylogeny. **a** Adult *D. pealeii* (image: Roger Hanlon). **b** Phylogeny of coleoid cephalopods derived from a single complete mitochondrial genome per species, with *Nautilus* as outgroup (not shown). Date ranges at nodes indicate minimum and maximum node ages in millions of years as estimated by a strict molecular clock. **c** Tissues collected from *D. pealeii* for RNA sequencing, classified as “Neural” (blue), “Non-Neural” (orange), and “Mixed” (purple) tissues. “Mixed” tissues correspond to axial nerve cord (ANC) and Retina (Ret) for containing heterogeneous cell types derived from neural and non-neural tissues. Blood (Blo—not pictured) and posterior salivary gland (PSG) were obtained from a non-reference *D. pealeii* individual.

To address these questions, we sequenced the genome of a single Atlantic longfin inshore squid *Doryteuthis* (formerly *Loligo*) *pealeii*, also known as the Boston market squid, and developed complementary transcript resources for analyzing RNA editing (Fig. 1a). Loliginid squid of the genera *Loligo* and *Doryteuthis* have played critical roles in the development of molecular and cellular neuroscience^13^. Famously, the mechanisms underlying the propagation of action potentials were deciphered using experimental preparations of loliginid giant axons, which transmit signals from the stellate ganglion to the muscular mantle^14,15^. More recently, these squid are models of cephalopod behavior^16–18^, development^19^, and cephalopod-derived biomaterials^20,21^. The value of these models has recently taken a leap forward with the development of CRISPR-mediated gene manipulation in *D. pealeii*, the first cephalopod to be edited^22^. Finally, loliginid squid are large pelagic predators that are important for fisheries in the Atlantic, Mediterranean, and Pacific Oceans and serve as sentinels of environmental change^23^.

## Results and Discussion

### Genome and gross gene content

We sequenced the large *D. pealeii* genome (4.6 Gb per haploid^24^) by combining single-molecular real-time long reads with deep short-read, mate-pair and chromatin conformation capture (“HiC”) sequencing (Methods). Over 10% of the genome is composed of the (AT)_*n*_ microsatellite, emphasizing the importance of unbiased long-read sequencing technology for cephalopods. We used genomic DNA from a single male individual to minimize the impact of the ~1.2% observed heterozygosity, which is modest for marine invertebrates but can introduce spurious redundancies unless alternate haplotypes are excluded from the primary assembly (“Methods”, Supplementary Note 1). Transcript data were collected from 27 tissues, 25 of which were isolated from the reference individual (Supplementary Table 3), facilitating the analysis of RNA editing. Our genome assembly totals 4.59 Gbp and comprises 46 long scaffolds (40–158 Mbp) (Supplementary Fig. 1) that we identify with chromosomes, matching the 2*N* = 92 karyotype that is shared by loliginids and related sepiids^25,26^. The assembly captures more than 96% of the known protein-coding gene complement (Supplementary Note 1).

To study genome evolution in coleoid cephalopods we also produced chromosome-scale assemblies of the Hawaiian bobtail squid, *Euprymna scolopes,* and the California two-spot octopus, *Octopus bimaculoides,* by combining new chromatin conformation capture sequences with previously reported draft genome assemblies^5,27^ (Supplementary Note 1). Figure 1b shows a phylogeny of representatives spanning several major cephalopod clades based on mitochondrial DNA (Supplementary Note 2), which accords with some previous studies^28^, although deep relationships among decapodiforms (squid and cuttlefish) have been notoriously difficult to resolve^29^. Using a molecular clock we estimate the *Euprymna-Doryteuthis* split to be ~100 million years ago (Mya), and the divergence of octopus from decapodiforms to be ~275 Mya (Fig. 1b, Supplementary Note 2), consistent with other studies^28,30^. The three chromosome-scale genomes analyzed here include representatives spanning the principal lineages of coleoid cephalopods.

We predicted 24,911 protein-coding genes in *D. pealeii* by combining extensive transcriptome data from 27 tissues with homology-based methods (Fig. 1c, Supplementary Table 3, Supplementary Note 3 and 4). Of these, 18,296 have detectable sequence similarity to protein-coding genes in other animals, comparable with the number found in octopus and bobtail squid (Supplementary Note 4). While most of these represent ancient genes found broadly across bilaterians, an additional 1597 *D. pealeii* genes have recognizable similarity only to genes from other cephalopods^9^. This nominally cephalopod-specific set includes several gene families that are present in both squids and octopuses (*e.g.*, reflectins) while others are restricted to squid (e.g., suckerins, histidine-rich beak proteins; Supplementary Table 4). These gene families related to cephalopod innovations are discussed below. Genes are irregularly distributed across chromosomes, with both regions of high gene density (a 19 Mb region with more than 50 genes per Mb) and long gene deserts (28 regions of at least 5 Mb with fewer than 1 gene per Mb) (Supplementary Fig. 2a). Gene density is positively correlated with LTR retrotransposons and proximity to chromosome ends, and negatively correlated with DNA transposons and simple repetitive sequence (Supplementary Fig. 2b), suggesting ongoing maintenance of distinct sub-chromosomal territories.

### Repetitive element landscape

The *D. pealeii* genome harbors an extensive complement of transposable and other repetitive elements, including numerous novel elements (Supplementary Note 4). Remarkably, while the squid and octopus genomes are all larger than typical invertebrate genomes, they have each expanded different families of transposable elements (Supplementary Table 5). SINEs dominate the repeat landscape in octopus^5^, but LINEs dominate the squids^27^, albeit from different classes in *Doryteuthis* and *Euprymna* (e.g., RTE-BovB in *Doryteuthis* and CR1-Zenon in *Euprymna*). The turnover of transposable elements is rapid within coleoid genomes, with the majority of elements (55%) showing limited (10%) divergence consistent with recent and possibly genus-specific expansion (Supplementary Note 4). Some repetitive elements are unevenly distributed across coleoid genomes, with subsets of chromosomes supporting the expansion and maintenance of distinct repeat classes (Supplementary Note 4). Since transposable elements have been implicated in the rewiring of gene regulatory circuits^31^, the difference in transposable elements across coleoid cephalopods could be one of the major drivers of genomic innovation among these diverse groups.

### Conserved synteny

**Fig. 2:**
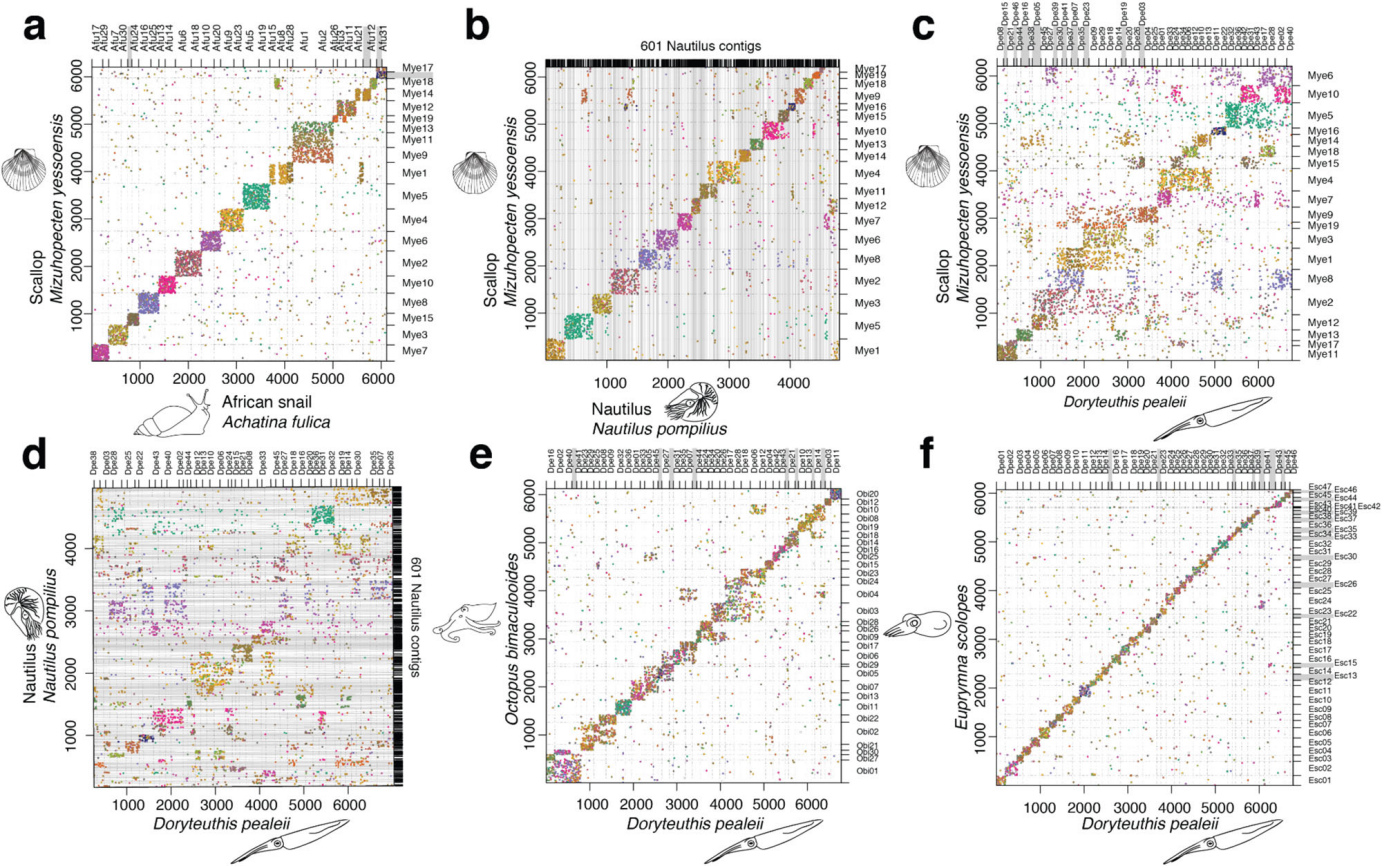
Conserved synteny across coleoid cephalopods. Dotplots of orthologous gene content. **a** The scallop *M. yessoensis* and the African snail *A. fulica*. The chromosomes of these two molluscs are conserved both in regard to each other and to their linkage group identities. **b** *M. yessoensis* and *N. pompilius*, a non-coleoid cephalopod, show conservation of macrosynteny between early branching cephalopods and other molluscs. **c** *M. yessoensis* and *D. pealeii* illustrate derived rearrangements in squid genomes. **d** Comparisons of *D. pealeii* and *N. pompilius* suggest chromosomal rearrangements occurred after the split between nautiloids and coleoids. **e** *D. pealeii* and *O. bimaculoides*. Squid and octopus chromosomes show higher levels of conservation. **f** *D. pealeii* and *E. scolopes*. The chromosomes show near 1:1 correspondence between the two squid species. Axes are labeled with chromosome or contig IDs and gene indices.

**Fig. 3:**
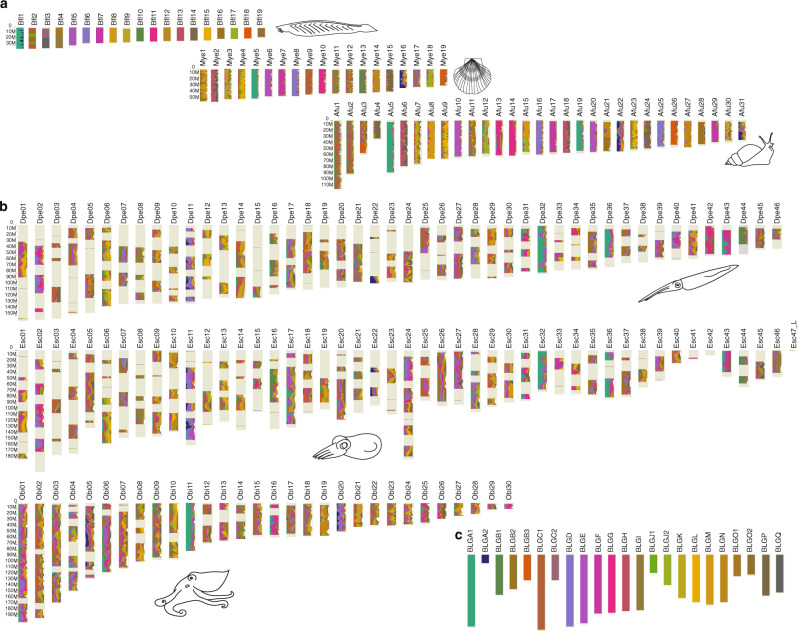
Bilaterian linkage group (BLG) orthologs on amphioxus, scallop, snail, and cephalopod chromosomes. **a** Top: Amphioxus (*B. floridae*) chromosomes correspond 1:1 to BLGs with some exceptions: Bfl1—chordate fusion of BLGA1 and A2; Bfl2—recent amphioxus fusion of BLGC1 and BLGJ1; Bfl3—recent amphioxus fusion of BLGC2 and BLGQ; Bfl4—recent amphioxus fusion of BLGO1 and BLGI. Middle: *M. yessoensis* chromosomes show some mixing of BLGs, but most chromosomes primarily correspond to one BLG. Bottom: *A. fulica* chromosomes follow similar patterns as *M. yessoensis* chromosomes, except that *A. fulica* underwent a whole genome duplication^94^ resulting in several duplicate chromosomes. **b** BLG orthologues on cephalopod chromosomes show extensive mixing of multiple BLGs throughout. Top: *D. pealeii* chromosomes. Middle: *E. scolopes* chromosomes. Bottom: *O. bimaculoides* chromosomes. **c** BLG color assignments.

**Fig. 4:**
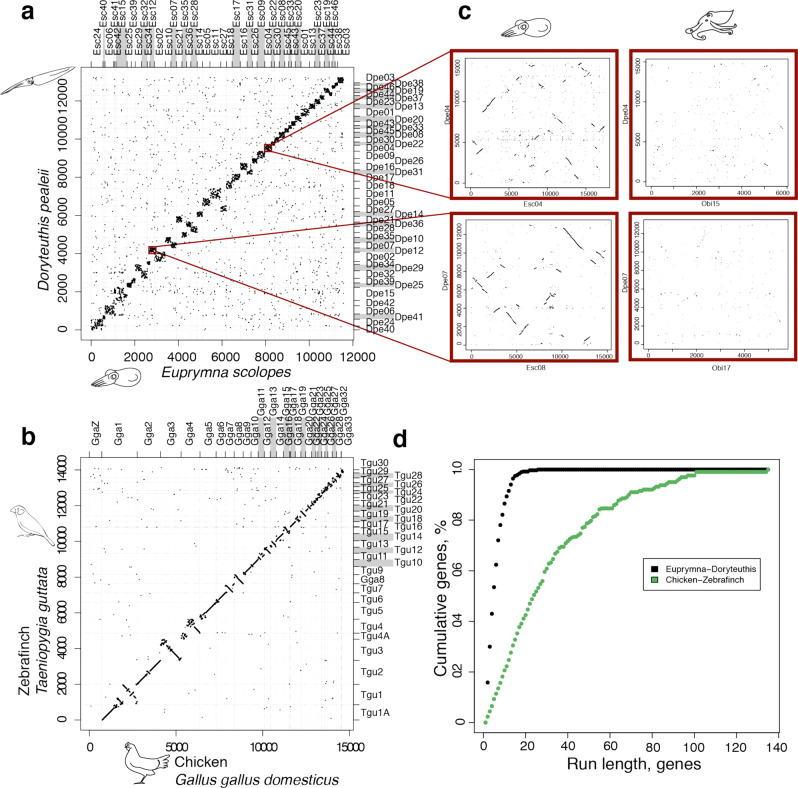
Disruption of colinearity in squid, but not bird, genomes. Mutual best hit dotplots between squids (**a**) and birds (**b**). **c** Megablast alignment in 10 kb windows of *E. scolopes* and *D. pealeii* (left) and *O. bimaculoides* and *D. pealeii* (right). The squid show some retention of colinearity while colinearity is lost between octopus and squid genomes. **d** Quantification of microsyntenic cluster sizes. Run length corresponds to the number of genes in a detected microsyntenic linkage (maximum number of intervening genes = 5), and cumulative genes (y-axis) corresponds to the total sum of genes in the run of a certain size or larger. This allows us to define an “N50” measure: 50% of squid genes are in microsyntenic runs of 4 or fewer genes and 50% of bird genes are in microsyntenic runs of 23 or fewer genes.

Despite 100 million years of divergence, and the differential transposable element expansions noted above, we find a remarkable near 1:1 correspondence between the chromosomes of *D. pealeii* and *E. scolopes*, reminiscent of the pattern observed between non-cephalopod molluscs (Figs. 2 and 3; Supplementary Note 5, Supplementary Fig. 3). This observation from genome sequences aligns with the finding that chromosome numbers in loliginid and sepiid squid are the same^25^. The karyotypic stability of squid is nominally similar to the karyotype stasis observed in birds^32^. Despite the nearly perfect 1:1 correspondence between loliginid and sepiolid squid chromosomes, we find extensive within-chromosome rearrangement (Fig. 4). This is a sharp contrast with birds, which have diverged over a similar time scale (~100 million years) but exhibit long-range intrachromosomal colinearity (Fig. 4).

Patterns of conserved synteny shared by squids and octopus relative to bivalve and gastropod molluscs, however, suggest a period of intense genome rearrangement prior to the split between the major coleoid groups (Figs. 2c, d, 3, Supplementary Fig. 4, Supplementary Note 5). This is confirmed by the observation that the recently published *Nautilus* genome^33^ largely preserves ancestral molluscan/bilaterian macrosynteny (Fig. 2d). Since sea scallop chromosomes show extensive conserved synteny with the chordate amphioxus and diverse marine invertebrates^34,35^, the ancestral molluscan chromosomes likely resembled scallop chromosomes in their gene content and organization. The organization of the *Nautilus* genome suggests that this ancestral state persisted in the earliest cephalopods. Coleoid cephalopod genomes, however, were extensively restructured. We find that genes linked together in scallop, clam, and *Nautilus* chromosomes are typically distributed across 1–9 squid chromosomes and 1–8 octopus chromosomes (Fig. 2c, d). This observation, combined with the simpler syntenic relationships between squid and octopus, demonstrates considerable interchromosomal rearrangement in the coleoid cephalopod stem lineage.

Our chromosome-scale assemblies allow us to compare the large-scale genome organization of two ancient coleoid lineages. Chromosome numbers of squid (*N* = 46) and octopuses (*N* = 30) are distinct but stable within each group^25,26,36^. We find that conserved syntenies (i.e., gene linkages) between octopus and squid allow their chromosomes to be put into simple groups that associate 1–4 squid chromosomes with 1–3 octopus chromosomes (Fig. 2e, f, Supplementary Fig. 3). These patterns imply limited rearrangement in the two lineages since their common coleoid ancestor. In a handful of cases, squid and octopus chromosomes can be put in 1:1 correspondence, indicating that these chromosomes (1) were present in the last common coloeid ancestor and (2) have been stable since that time (Supplementary Table 10).

From these patterns of conserved synteny we infer that the proto-coleoid chromosomes arose from the bilaterian-like linkage groups present in the most recent common ancestor of cephalopods, bivalves, and gastropods by a process of fragmentation and mixing that produced the novel combinations seen in coleoids. The net effect of these rearrangements was to reorganize the 21–22 ancestral molluscan chromosomes into at least 32 ancestral coleoid cephalopod linkage groups. We define these ancestral coleoid groups as sets of genes that are (1) syntenic in both coleoid lineages but (2) show the least amount of mixing relative to other bilaterians (Supplementary Note 5). Of these ancestral coleoid linkage groups, 28 have been retained in *Doryteuthis* without subsequent fusion (although chromosome fragmentation led to them being spread over 40 chromosomes) and 18 in octopus (spread across 20 chromosomes). The remaining four ancestral coleoid linkage groups underwent fusions followed by mixing (Fig. 3, Supplementary Table 9). Surprisingly, our results reveal that octopus chromosomes, despite reduced numbers, retain fewer ancestral bilaterian linkage group (BLG) fission products than do squid. Accordingly, we find more BLG mixing on the octopus chromosomes, suggesting secondary, lineage-specific, fusions (Fig. 3, Supplementary Fig. 5, Supplementary Table 9). This comparative analysis suggests that the ancestral coleoid syntenies more closely resembled those found in contemporary squid such as *D. pealeii* and *E. scolopes*, with many octopus chromosomes formed by fusions followed by intrachromosomal scrambling of gene order.

Intriguingly, the stem lineages of both coleoid cephalopods and jawed vertebrates each experienced analogous periods of intense genomic rearrangement. The jawed vertebrate rearrangements occurred in the aftermath of the early vertebrate genome duplications^34^. In contrast, the rearrangements in the coleoids was not accompanied by genome duplication, which we can rule out^5^ based on the scarcity of unlinked duplicates in the squid and octopus genomes. Our findings suggest further study of the connection between chromosomal “big bangs”—whether due to extensive rearrangement as in cephalopods, or whole-genome duplication as in vertebrates—and the evolution of novel body plans, complex nervous systems, and other adaptations.

### Large gene clusters

Cephalopod genomes are known to encode an expanded repertoire of protocadherin and C2H2 gene families^5,27,37,38^. Using our chromosomal sequences for *D. pealeii*, *E. scolopes*, and *O. bimaculoides* we assessed the full extent of these gene families and their genomic organization across coleoid cephalopods. The protocadherin (PCDH) gene family is larger in coleoids than in other bilaterians, and is even larger in *D. pealeii* (288) than in *O. bimaculoides* (168)^5,37^ or *E. scolopes* (220) (Supplementary Fig. 6a)^27^. In vertebrates, PCDHs are homophilic neuronal cell adhesion molecules^39,40^, and they may play a role in coordinating the development of the large nervous systems of coleoids.

**Fig. 5:**
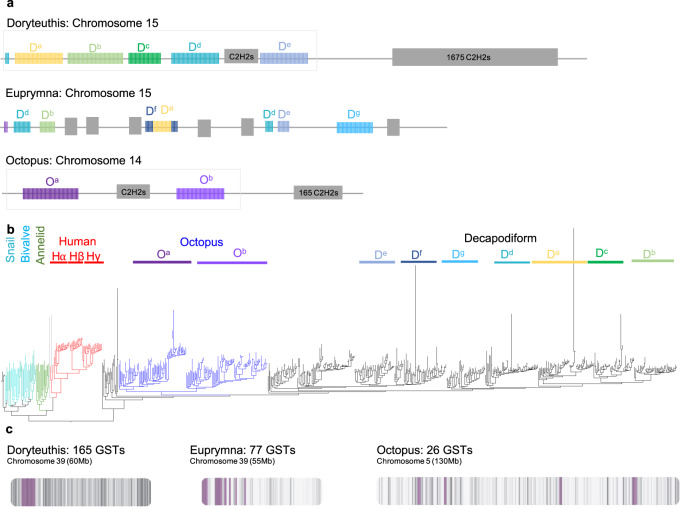
Expansion of gene families. **a** Protocadherin gene clusters in cephalopod genomes. The protocadherin- and C2H2-rich chromosomes for *D. pealeii, E. scolopes*, and *O. bimaculoides* are shown to scale. 285/288 *D. pealeii* *PCDH*s are located within a 50 Mb cluster on chromosome 15 (box). Of these, 163 are found in 5 tight subclusters of 40 (D^a^, yellow), 37 (D^b^, grass green), 20 (D^c^, green), 36 (D^d^, teal), and 30 (D^e^, blue) genes. All but the D^e^ are facing in the same transcriptional direction. *E. scolopes* also demonstrates multiple clusters of *PCDH*s spanning chromosome 15. The orthologous chromosome in *O. bimaculoides* contains 149 of 168 *PCDH*s found in the genome, with two notable subclusters of 34 and 27 genes. Major clusters of C2H2 genes are noted in gray. **b** Phylogeny of coleoid (octopus: blue, decapodiform in black), snail (*Lottia gigantea*, teal), bivalve (*Crassostrea* *gigas*, sky blue), annelid (*Capitella teleta*, green) and human (red) *PCDH*s demonstrates lineage-specific expansions. A handful of very long branches in the decapodiform protocadherins correspond to truncated sequences that may represent pseudogenes. Notably, genomic clusters (indicated above the phylogeny by different color bars) also cluster on the tree. **c** Arrangement of S-crystallins/Glutathione S-transferases (GSTs) in cephalopod genomes. Purple bars indicate the location of GST genes, gray gradient indicates gene density. *D. pealeii* has 139 *GST*s in a single cluster spanning 60 Mb on chromosome 39. The orthologous chromosome in *E. scolopes* contains 77 *GST*s distributed in multiple clusters, while in octopus, 26 *GST*s are found spanning chromosome 5.

Protocadherin expansions form multi-megabase arrays on single, orthologous chromosomes in the three coleoid genomes (Fig. 5a). Almost all of the *D. pealeii* PCDH genes (285/288) are encoded by a large 50 Mb cluster on chromosome 15, with more than half of these genes organized as five sub-arrays of closely related, multi-exonic genes oriented in the same transcriptional direction (Fig. 5a, b). We also identified fragmentary reading frames with some sequence similarity to the protocadherins throughout these clusters, which may represent pseudogenes. The orthologous *O. bimaculoides* chromosome 14 contains a 43 Mbp cluster organized into two sub-arrays of closely related genes. Although these chromosomes are orthologous, *D. pealeii* and *O. bimaculoides* PCDH genes themselves form distinct phylogenetic sub-families, suggesting a combination of ongoing lineage-specific gene duplication and homogenization through gene conversion (Fig. 5b). Protocadherin genes in *E. scolopes* and *D. pealeii* are phylogenetically interspersed but include some subclusters unique to each species. Unlike vertebrates, cephalopods do not generate *PCDH* diversity by alternative splicing where diverse sets of multiple first exons are spliced to shared final exons^40^. Instead, the *PCDH* diversity in coleoid cephalopods arose convergently through a distinct mechanism of full gene duplications.

The complement of C2H2 zinc finger transcription factors is also dramatically expanded in all three coleoid genomes (Supplementary Fig. 6c), which was suggested in findings from sub-chromosomal draft coleoid genomes^5,27^. The *D. pealeii* genome encodes a staggering 2785 C2H2-domain-containing genes, with the majority (1675, or 60%) contained on chromosome 15, on the opposite end of the chromosome from the *PCDH* supercluster. The orthologous chromosome in *Octopus* is similarly arranged, but with many fewer C2H2 genes (165, or 9% of the total). *Euprymna* chromosome 15 contains 201 C2H2 genes, but they are more interspersed with the *PCDH* sub-arrays (Fig. 5a). In contrast to the genomic organization of protocadherins, the C2H2 genes in *D. pealeii* do not form phylogenetically-related subclusters.

In addition, we found an extensive expansion of S-crystallins, which are related to the glutathione S-transferases and constitute the majority of crystallins characterized in squid lenses^41,42^. A moderate expansion in decapodiforms had been previously detected^43^. Here we report 139 S-crystallin genes located in a single, tight cluster spanning 5.5 Mb on chromosome 39 in the *D. pealeii* genome (Fig. 5c). This is a larger expansion than has been described in *E. scolopes* (77) and is a considerably increased complement relative to the 27 S-crystallin genes of the *O. bimaculoides* genome. In contrast, the *O. bimaculoides* genome contains a clustered expansion of 26 acetylcholine receptor-like genes on chromosome 15 that are expressed in the suckers^5^ and have recently been shown to contribute to chemosensory reception^44^. *D. pealeii*, however, encodes only seven of these atypical subunits, of which five are tightly clustered on chromosome 4 (Supplementary Fig. 6d).

While the phenotypic role of some gene family expansions is little understood, several expansions that we highlight in these cephalopod genomes do have important roles in other animals. The diversity of protocadherins, for example, is important in establishing neuronal self-avoidance in vertebrate brains^40,45^. In vertebrates, the expansion of protocadherins translates to cell-surface diversity in a role analogous to DSCAM in flies, and a reduction in this diversity results in inappropriate neuronal connections and cell death^46–48^. Ecdysozoans lack this gene family entirely, and ambulacrarian genomes only encode a single protocadherin (Supplementary Fig. 6a), suggesting that the expansion of the size of this gene family was required for vertebrate neuronal diversity. While we make no claims about their function, our work demonstrates a similar correlation, with a large diversity of protocadherins expressed in the elaborate coleoid brains (Supplementary Fig. 6b). By contrast, only a handful of protocadherin genes are found in their spiralian relatives.

The diversity of glutathione S-transferases in squid has been demonstrated to play a role in the formation of the refractive gradient of their lens, with proteins with short linkers in the center, and those with longer linkers at the periphery^49^. While a diversity of these sequences had been suggested from previous RNA-seq analyses^50^, here we demonstrate a far greater expansion than what had been previously detected, as well as a striking genomic arrangement.

### RNA editing

A striking feature of coleoid cephalopods is their extensive editing of messenger RNAs by enzymes that convert specific adenosines (A) to inosines (I). Since inosine is interpreted by the translational machinery (and in cDNA sequencing) as a guanosine (G), mRNA editing can lead to “recoding,” which allows a static genome to dynamically encode a diverse proteome^51,52^. While A-to-I mRNA editing occurs in other animals, it has been reported to be several orders of magnitude more prevalent in coleoid cephalopods^9,10^. Case studies of specific neuronal genes have argued that mRNA editing in cephalopods can be adaptive^7,9,53,54^, although editing is influenced by diverse evolutionary forces and non-adaptive explanations have also been proposed^55^. The frequency and tissue-specificity of coeloid mRNA editing are poorly characterized in part due to the lack of a complete, high-quality reference genome against which transcriptomes can be compared.

**Fig. 6:**
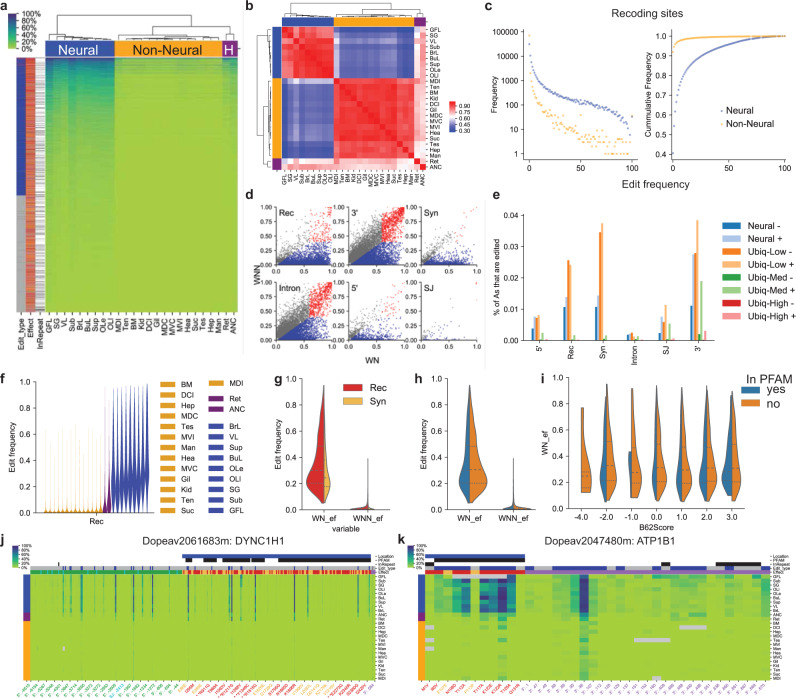
RNA editing profiles in *D. pealeii*. **a** Edit frequencies of target sites (y-axis) per tissue sample (x-axis) from constitutively expressed edit sites. **b** The correlation matrix illustrates how squid tissues cluster by their edit frequencies. Clustering of tissues shows distinct groups, neural tissues to the left (blue), non-neural tissues in the center (yellow), and mixed tissues on the right (heterogeneous - “H”, dark purple): retina (Ret) and axial nerve cord (ANC). 13,578 constitutively expressed sites that have more than 3 reads in each of the samples with at least 5% or more edit frequency in at least one sample (**a** and **b**). **c** Frequency distribution of recoding edit sites discriminated by neural (blue) and non-neural (orange) samples. The majority (54%) of the recoding edit sites in neural samples have an edit frequency below 1%; in contrast, most of the recoding sites (94%) in non-neural samples are below 1%. **d** Scatterplot of the weighted average edit frequencies of neural samples (WN) against the weighted average edit frequencies of non-neural samples (WNN) classified by edit type: recoding (Rec), synonymous (Syn), intronic (Intron), splice junction (SJ), or in the 5′ or 3′ UTR. The weighted averages were used to classify edit sites as: *Neural* with differential editing between neural and non-neural samples where the ratio between WN and WNN is above 2.75 (blue); *Ubiquitous Low* with edit frequencies below 5% (light gray); *Ubiquitous High* with editing frequency rates >60% for neural and >40% for non-neural tissues (red); *a*nd *Ubiquitous Medium* with edit frequencies between 5–40% in WN and 5–60% in WNN (gray). **e** 197,549 sites with at least 10 reads of depth in neural and non-neural samples classified by genic locations and overlap with repetitive sequence (as indicated by the + and −). Coding edits are found predominantly in *Neural* and *Ubiquitous Low* edit types while repetitive sequences are frequently edited in the 3′ UTR, regardless of edit type. **f**–**i** Analysis of edit frequencies of neural-type edits that are robustly edited (>25% edit frequency in at least one sample). **f** The edit frequency per tissue highlights the GFL as the tissue with the highest distribution if edit frequency. Side-by-side comparison of weighted edit frequencies  of **g** recoding and synonymous sites, and **h** sites overlapping conserved protein domains. **i** Same as (**h**), showing the WN values segregated on the x-axis by the amino acid substitution score. Heatmap of RNA editing profiles for the constitutively expressed Dynein Cytoplasmic 1 Heavy Chain 1(DYNC1H1) gene (**j**), and the ATPase Na+/K+ Transporting Subunit Beta 1 (ATP1B1) gene (**k**), which is expressed in all neural tissues.

To characterize organismal patterns in *D. pealeii* mRNA editing we developed a comprehensive map of edited sites from a diverse set of 24 neural and non-neural transcriptomes (Fig. 6a, b, Supplementary Table 3). Since the reference genome and transcriptomes were obtained from the same individual, we could readily differentiate edited sites in transcripts from heterozygous A/G sites in the genome. Our study complements previous analyses that use more restricted tissue sampling and localized genic assemblies^9,10^. We computed tissue-specific edit frequencies, that is, the fraction of transcripts that are read as G (corresponding to inosine in mRNA) relative to the genomically encoded A (“Methods”, Supplementary Note 7).

We found a total of 590,165 A-to-I edited sites genome-wide, the majority of which are edited at low frequency (Fig. 6c, Supplementary Figs. 7c and 8a). Out of those, 205,618 sites demonstrated robust editing with an edit frequency above 25% in at least one sample (Supplementary Table 11). There are 11,841 genes edited in the genome (Supplementary Table 12). Nearly a quarter of robustly edited sites (56,520 out of 205,618) are found in 5905 genes, including both recoding (15,293) and synonymous (5528) sites (Table 1). RNA editing is also enriched in 3′ UTR and coding sequences in comparison with 5′ UTR and introns relative to the number of potentially editable adenosines of these genic features (Supplementary Table 13). We also found 376,148 A-to-I edited sites in transcribed sequences other than annotated protein-coding genes. Most sites in untranslated regions overlap annotated repetitive elements (Supplementary Table 14, Supplementary Fig. 8b), consistent with a role for A-to-I editing in inhibiting retrotransposon activity^56,57^.

**Table 1:** The number of ADAR target sites found in different gene features subclassified by robustness and by edit type.

		Edit type						
Robustness	Edit_type	5′	Rec	Syn	SJ	Intron	3′	Total
>25% editing (Robust)	Neural	1646	13,965	4854	78	8728	9467	38,738
	Ubiq-High	56	206	28	10	1049	1022	2371
	Ubiq-Low	101	414	231	6	537	609	1898
	Ubiq-Med	322	519	355	22	4924	5327	11,469
	Other	82	189	60	3	1500	210	2044
	Total robust	2207	15,293	5528	119	16,738	16,635	56,520
<25% editing (Not robust)	Neural	1024	8303	4641	42	3568	6175	23,753
	Ubiq-Low	4368	53,182	30,971	268	11,099	30,273	130,161
	Ubiq-Med	60	139	123	11	642	1518	2493
	Other	103	165	71	4	605	142	1090
	Total not robust	5555	61,789	35,806	325	15,914	38,108	157,497
TOTAL		7762	77,082	41,334	444	32,652	54,743	214,017

Unclassified edits belong to sites with insufficient cumulative read depth (<10 reads).

Patterns of mRNA editing are highly correlated across tissues, with neural and non-neural samples forming distinct groups (Fig. 6). Correspondingly, we find two types of sites: those that are edited predominantly in neural tissues (neural edits), and those that are edited at comparable frequencies across all tissues (ubiquitous edits; Fig. 6a, d, Supplementary Note 7). These two categories differ not only in their tissue-specific editing, but also in overall editing frequency. The great majority of ubiquitously edited sites are edited only at low frequency; 78% of the edit sites have an average edit frequency below 2% (Supplementary Fig. 7). Low-frequency recoding edits may not be adaptive but rather may be a byproduct of ADAR activity near targeted (and more robustly edited) sites (Fig. 6j, k, and Supplementary Figs. 10–12). While ubiquitous-type editing frequencies were similar across all tissues, neural-type sites showed differential editing among neural tissues, with giant fiber lobe (GFL) having the highest rate of editing among the neural tissues studied (Fig. 6a, c, f).

Neural and ubiquitous edit sites also have distinct distributions across gene bodies (Fig. 6d, e). Neural edits are predominantly found in coding sequences; of such sites 70% are recoding and 30% are synonymous. In contrast, ubiquitously edited sites are predominantly found in 3′ UTRs and introns, and tend to overlap annotated repetitive sequences. Due to these differences between neural and ubiquitous sites, the vast majority of robustly edited recoding sites are of the neural type (91%), so that robust recoding outside of the nervous system is relatively rare (Table 1).

While the potential for recoding edits to alter protein function suggests that recoding sites should be correlated with functional domains or conserved sequences, we did not find any consistent functional signal. Specifically, we did not find significant differences in recoding edits in coding regions within or outside of conserved protein domains (Fig. 6h, Supplementary Fig. 9b), nor did we find preference for recoding edits in or outside transmembrane domains (Supplementary Fig. 9c). We also did not find differences in the nature of amino acid substitution in recoding, as determined by the Blosum62 score (Fig. 6i, Supplementary Fig. 9b, d). These bioinformatic observations suggest that the impact of recoding on protein function may be subtle and specific to each recoded protein, as found for specific potassium channels^7,9,58^.

A notable example of mRNA editing in mammals is the GRIK family of ionotropic glutamate (kainate) receptors, which are involved in short-term synaptic plasticity^59^. In mammals, two of the five *GRIK*s are shown to be edited by ADAR; the edited *GRIK2* gene products regulate receptor permeability^60^. In squid, the three GRIK orthologs are also neurally expressed but are far more extensively edited than their mammalian counterparts, primarily within known functional domains. Only one of the three well-characterized mammalian *GRIK2* editing sites, however, is genomically conserved and edited in *D. pealeii* (Tyr512Cys in squid; Tyr571Cys in human and mouse) (Supplementary Fig. 11). In addition to genes with clear neural function, we also find neural-specific editing in genes with broad biological function, such as dynein (DYNC1H1) and the ATPase Na+/K+ Transporting Subunit Beta 1 (ATP1B1) (Fig. 6j, k). Both transcripts harbor recoding edits that result in nominally conservative amino acid changes. The importance of extensive squid editing in both neural-specific and broadly expressed genes will need to be addressed in comprehensive functional studies.

**Fig. 7:**
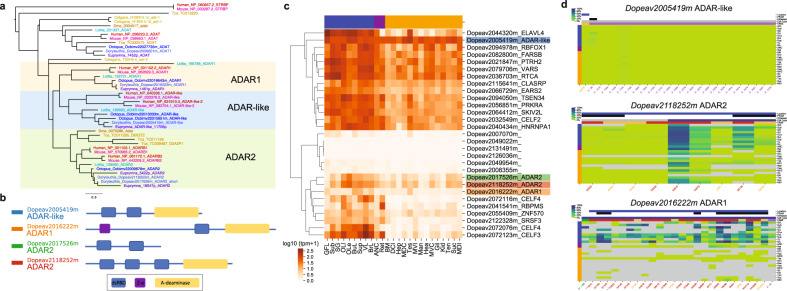
Expression and editing of ADAR transcripts. **a** Phylogenetic tree of ADAR homologs. The colors highlight ADAR1, ADAR2, and ADAR-like families. **b** Cartoon representation indicating conserved domains present in squid ADAR proteins: double-stranded RNA binding Domains (dsmr), Z-binding domain binds (Z-a), Adenosine deaminase (A-deaminase). **c** Expression of genes with PFAM domains that interact with RNA that are enriched in neural samples. Tissue abbreviations as in Fig. 1 and color code in top row as in Fig. 6. **d** mRNA editing profile of ADAR genes.

The genes that encode A-to-I editing enzymes (Adenosine Deaminase Acting on RNA, ADAR) are broadly transcribed in *D. pealeii* (Fig. 7), implying that transcriptional regulation is not sufficient to explain neural-specific editing. Notably, the *ADAR1* and *ADAR2*^61^ mRNAs are themselves extensively edited in the nervous system, possibly allowing feedback regulation. *ADAR1* demonstrates at least 20 recoding edits affecting 17 amino acids in *D. pealeii*, 6 of which are conserved with *O. bimaculoides*. Editing in *ADAR2* is dominated by two recoding sites that are predominantly neurally edited. Predominantly neural editing patterns are also observed among the transcripts of RNA binding proteins (Supplementary Fig. 10) including the squid CELF2 gene, which is known to associate with RNA editing enzymes in mammals^62^. Taken together these observations suggest regulation of ADAR activity at multiple levels.

To explore the evolutionary turnover of edit sites across loliginids, we shotgun sequenced the genomes of the congeneric Pacific market squid *Doryteuthis opalescens* (~28 Mya since common ancestor with *D. pealeii*, Fig. 1) and the more distantly related Japanese spear squid *Heterololigo bleekeri* (*Hbl*, ~48 Mya) and compared these sequences with the *D. pealeii* genome (Supplementary Note 7). Edited sites in *D. pealeii* that overlap coding regions are generally highly conserved compared with other adenosines (Supplementary Table 15), indicating that the potential for editing at these sites has been preserved since at least the origin of loliginids, consistent with previous comparisons among more distantly related coleoids^9^. Relatively few new editing sites have arisen in *D. pealeii* since its divergence from *D. opalescens (Dopal)*, i.e., positions that are A-to-I edited in *D. pealeii* but are not genomic adenosines, and therefore not edited, in the other two species. These include 114 edits of the neural type, 42 of which are recoding, that appeared in the *D. pealeii* lineage (Supplementary Table 16). Thus, while the evolution of editing is ongoing, it appears to have slowed relative to an original burst of new mRNA editing in the coleiod lineage.

Our analysis of a comprehensive set of tissue-specific transcriptomes compared with a high-quality reference genome complements previous studies^7,9,10^ and implicates broader roles for RNA editing beyond recoding. While we observe elevated rates of editing in the nervous system, many of the editing sites are not obviously associated with neuronal functions and include many “housekeeping” genes (Fig. 6e). Indeed, the vast majority of edits occur outside the nervous system and are enriched in noncoding regions. In vertebrates extensive editing of Alu and LINE elements has been described and, among other functions, is suggested to have roles as an additional line of defense against transposable element proliferation^57,59,63,64^. A similar role may be one of their major yet unexplored functions in cephalopod genomes.

### Cephalopod innovations and the genome

**Fig. 8:**
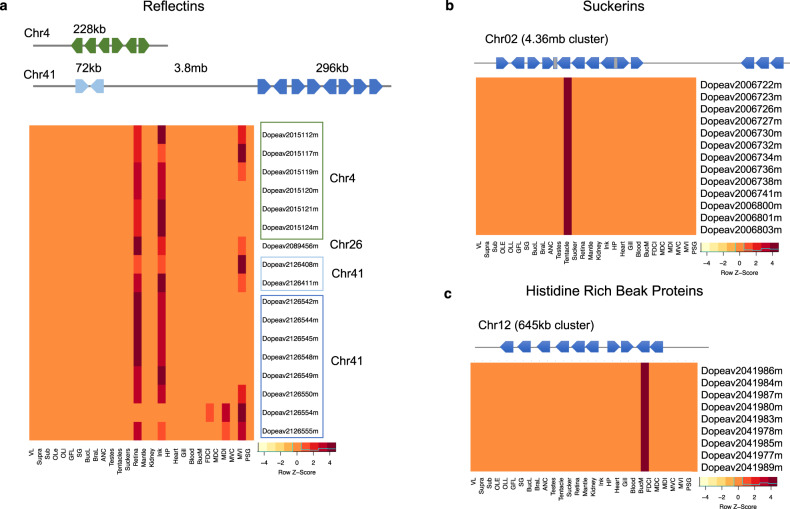
Cephalopod-specific gene families in the *D. pealeii* genome. **a** Reflectins in the *D. pealeii* genome. Top: three clusters of reflectins were identified on two chromosomes, with a single reflectin found on chromosome 26 (not shown). Bottom: Reflectin expression profiles across *D. pealeii* transcriptomes indicate these genes are deployed in iridescent tissues, including the iridophore layer of the skin, the tissue surrounding the eye (retina), and the ink sac. Cells are colored according to standard deviation from mean expression levels. **b** Suckerin genes in *D. pealeii*. Top: the *D. pealeii* genome contains 13 suckerin genes distributed in two clusters on chromosome 2. Bottom: heatmap of the expression profiles of the suckerins across *D. pealeii* transcriptomes demonstrate that the suckerins are most highly expressed in the club of the tentacle. **c** Histidine-rich beak proteins in *D. pealeii*. Top: Cluster of 10 histidine-rich beak proteins on chromosome 12. Bottom: Heatmap of expression profiles of histidine-rich beak proteins in *D. pealeii* transcriptomes demonstrate high expression in the buccal mass. Abbreviations as in Fig. 1 except: BucL buccal lobe, BraL brachial lobe, BucM buccal mass, FDCI dorsal fin skin.

Finally, we identified several families of taxonomically restricted genes that are specifically associated with some of the morphological and behavioral innovations of coleoids. Well-known among these are the reflectins, which play a role in the structural coloration and iridescence of cephalopod skin (Fig. 8A)^65^. We found 17 reflectins distributed in three tight clusters in the *D. pealeii* genome. We also identified two closely linked clusters of suckerins, a gene family associated with the sucker ring teeth in squid and cuttlefish (Fig. 8B)^66^. *D. pealeii* chromosome 2 encodes 13 suckerins, all of which are highly expressed in the tentacle, comparable with the 16 suckerin genes we identify in *E. scolopes*. The *D. pealeii* genome also contains a cluster of 10 histidine-rich beak proteins that are expressed in the buccal mass (chromosome 12, Fig. 8C). Histidine-rich beak proteins are thought to play a role in the mechanical properties of squid beaks^67^. While the reflectins are found across coleoids, we were only able to identify the suckerins and the histidine-rich beak proteins in *D. pealeii, E. scolopes*, and *Architeuthis dux*^68^, but not *O. bimaculoides*, suggesting that these novel gene families are associated with the evolution of decapodiform morphological innovations. We also saw additional arrays of genes in *D. pealeii* that have no similarity to other known proteins. These arrays likely represent additional clade-specific gene families (Supplementary Table 4).

Our study shows that cephalopod biology is paralleled by the unique evolutionary history of their genomes. Comparative genomic analyses using chromosome-scale assemblies of the two main coleoid cephalopod lineages highlight a balance between innovations at different levels of genome organization. While some genomic characters such as specific gene family expansions have evolved convergently with vertebrates, other features such as overall genome organization are strikingly different from other animals. In particular, we reveal that the coleoid ancestor has undergone a genome-wide reshuffling of ancestrally distinct chromosomes. While the outcome is generally analogous to fusions observed in the vertebrate lineage, the mechanism does not rely on whole-genome duplication, which was absent from the cephalopods. This reorganization was restricted in time since much of the modern-day karyotype is preserved among the main coleoid lineages. Within coleoids, however, lineage-specific evolution seems to have been governed by novel gene formation, independent expansions among key gene families, and substantial RNA editing. Together we posit that understanding this mode of genome evolution—the evolutionary decoupling of different genomic characters—will be key to understanding the genomic basis of cephalopod organismal innovations.

## Methods

### De novo assembly of the *Doryteuthis pealeii genome*

All work was performed in compliance with the EU Directive 2010/63/EU on cephalopod use and AAALAC guidelines on the care and welfare of cephalopods^69–71^. We sequenced the genome of *D. pealeii* using a whole genome shotgun approach that combined long single-molecule PacBio reads with short, high accuracy paired-end Illumina data (Supplementary Table 1). Genomic DNA for all shotgun sequencing was derived from a single male collected in October 2015 by otter trawl from Vineyard Sound, by the Marine Resources Center at the Marine Biological Laboratory, Woods Hole, MA. The same individual was used for almost all RNA sampling. For genomic DNA isolation, testis tissue was quickly dissected, flash-frozen on liquid nitrogen and stored at −70 °C. Genomic DNA (gDNA) was extracted by homogenizing and digesting testis tissue with proteinase K at 55 °C overnight. A 1/3 volume of 5 M NaCl was gently mixed in, and the homogenate was spun at 1000 × g for 5 min to precipitate the protein. The supernatant was transferred to a new tube and 2 volumes of ice-cold 100% ethanol was added. High molecular weight gDNA was spooled, washed with 75% Ethanol, and resuspended overnight in nuclease-free water (Sigma) at 4 °C and stored at −70 °C until use. We assembled the *D. pealeii* genome using a hybrid approach, aiming for a single representative haplotype across the genome (Supplementary Note 1). We also generated shotgun reads for *D. opalescens* and *H. bleekeri* (Supplementary Note 2).

### Chromosome-scale assembly of the *Octopus bimaculoides genome*

To produce a chromosome-scale assembly for *O. bimaculoides*, we integrated new HiC datasets (deposited under Bioproject PRJNA808169) with the previously published shotgun assembly^5^ (Supplementary Note 1).

### Chromosome-scale assembly of the *Euprymna scolopes genome*

The *E. scolopes* assembly was generated using HiC data and assembled with Lachesis^72,73^. Scaffolds of 50 kb and longer from the publicly available^27^ assembly were used together with aligned Hi-C reads.

### Molecular phylogeny and dating

We inferred molecular phylogeny and divergence times of nineteen species that represent the major cephalopod lineages using mitochondrial protein-coding genes (Supplementary Table 2). We retrieved open reading frames using the stand-alone ORFfinder (https://www.ncbi.nlm.nih.gov/orffinder/) and mitochondrial protein-coding genes were annotated considering the best hit between amino acid sequences of our target species with those of *Idiosepius* (accession number KF647895) using BLASTP. Each protein-coding gene was aligned codon-based using MUSCLE 3.8^74^ implemented in AliView^75^. We performed maximum likelihood analyses of concatenated sequences in IQ-TREE^76^ with the best model and partition scheme selected by ModelFinder^77^, and 1000 replicates of ultrafast likelihood bootstrap^78^.

We estimated the age of each node assuming a strict clock with the Langley–Fitch method in r8s 1.8^79^. We rooted the tree generated in IQ-TREE and fixed the age of two internal nodes, one for the crown Cephalopoda to 328 and 254 Mya, and the other for the divergence between the Vampyromorphida and Octobrachia to 276 and 206 Mya. These ages correspond to the maximum and minimum age estimations using transcriptome data from Supplementary Figure 4 in^28^.

### Transcriptome Sequencing

We generated transcriptomes from 28 different tissues to aid gene prediction and to enable expression and RNA editing analyses (Supplementary Table 3). All tissues except for the posterior salivary gland and blood samples were obtained from the same adult male specimen that provided genomic DNA for shotgun sequencing. Tissues harvested were quickly dissected and flash frozen on liquid nitrogen with a small amount of Trizol (Invitrogen). Samples were stored at −70 °C and RNA was isolated using Trizol following the manufacturer’s instructions.

RNA integrity was analyzed with a Bioanalyzer 2100; only samples with clean rRNA peaks and little to no degradation were used. Total RNA was polyA-selected and directionally sequenced at the University of Chicago Genomics Facility on an Illumina HiSeq2000 per manufacturer’s instructions, generating paired-end 2 × 100 bp reads with an insert size of ~300 bp. These reads are deposited under BioProject PRJNA641326.

### Protein-coding gene annotation

We annotated protein-coding genes of the *D. pealeii* genome using the DOE Joint Genome Institute (JGI) annotation pipeline (img.jgi.doe.gov/docs/pipelineV5/). RNA-seq data from 28 tissues (Supplementary Table 3; PRJNA641326) were aligned to the genome and assembled on-genome into transcripts by PERTRAN^80^. Assembled transcripts were aligned to the genome using PASA^81^, and PASA alignments, along with exonerate alignments of the proteomes of *O. bimaculoides, Aplysia californica, Crassostrea virginica, Homo sapiens, Xenopus tropicalis, Lottia gigantea*, and Swissprot eukaryotes (downloaded November 2017). The alignments and peptide homology sequences of the transcript assemblies and the peptides were submitted to GenomeScan^82^ and Fgenesh+^83^ for gene predictions. A best prediction per locus was selected and used to add UTR, to correct intron/exon boundaries with transcript data, and to add additional splice isoforms with PASA.

### Repetitive landscape

Repeats were annotated using the RepeatModeler (2.0)^84^ and RepeatMasker (open-4.0.7)^85^ pipelines.

### Gene family evolution

Gene families of particular interest were manually curated and analyzed as described in^5^. Briefly, we searched for genes of interest in the *D. pealeii* genome and transcriptome assemblies using BLASTP and TBLASTN searches. Candidate genes were verified using BLAST and Pfam. Genes identified in the *D. pealeii* genome were confirmed and extended using the transcriptomes, and multiple gene models that matched the same transcript were combined. The identified sequences from *D. pealeii* and other bilaterians (*H. sapiens*, *Mus musculus, Drosophila melanogaster, Tribolium castaneum, Caenorhabditis elegans, Capitella teleta, C. gigas, L. gigantea*, and *O. bimaculoides)* were aligned using either MUSCLE^74^ or CLUSTALO^86^. Phylogenetic trees were constructed with FastTree2^87^, using full-length sequences, and visualized with Figtree (A. Rambaut, http://tree.bio.ed.ac.uk/software/figtree/).

### Mutual best hit orthology

Reciprocal BLASTP (version 2.10.0+^88^) for *E. scolopes, D. pealeii, O. bimaculoides*, and *M. yessoensis* was run against *B. floridae* and mutual best hits (MBHs, e-value cutoff = 1e-2) were combined to form 6,821 core orthologs. Orthologs of this core set were then identified in the remaining species (Supplementary Table 7) by computing their mutual best hits to *B. floridae* and merging with the 6,821 gene core set. A custom script was used to extract the genomic locations of those orthologs from their genome annotations. Shared orthologs between species were clustered with Euclidean clustering and plotted in R as dotplots. The number of CephLGs was inferred via counting of orthologous chromosomes in *D. pealeii* and *O. bimaculoides* with the least number of BLG combinations

### RNA editing analysis

#### Transcriptome variant calls

Transcriptomes obtained from tissues originating from the genome-reference individual were used for the analysis (Specimen A, Table S3). RNA-seq reads were aligned against the squid genome with the STAR aligner 2.5.3a^89^. The first round of RNA-seq alignments followed the following parameters: ‘-outSAMtype BAM SortedByCoordinate -runThreadN 8 -chimOutType SeparateSAMold -chimSegmentMin 20 -chimJunctionOverhangMin 20 -outSAMstrandField intronMotif -alignSoftClipAtReferenceEnds No -outSAMmapqUnique 255 -outFilterMultimapNmax 1 -outReadsUnmapped Fastx -sjdbFileChrStartEnd /projectb/scratch/mitros/squid/alnV2/star/sjdb.20.txt -sjdbGTFfile Dpealeiiv2.gtf’, and the second round: ‘-outSAMtype BAM SortedByCoordinate -runThreadN 8 -chimOutType SeparateSAMold -chimSegmentMin 20 -chimJunctionOverhangMin 20 -outSAMstrandField intronMotif -alignSoftClipAtReferenceEnds No -outSAMmapqUnique 255 -outFilterMultimapNmax 1 -outReadsUnmapped Fastx -outFilterMismatchNmax 999 -winBinNbits 10 -outFilterMismatchNoverLmax 0.5 -outFilterMismatchNoverReadLmax 10 -alignMatesGapMax 55000 -outFilterScoreMin 100 -outFilterIntronMotifs RemoveNoncanonical -outFilterMatchNminOverLread 0.2 -sjdbFileChrStartEnd sjdb.20.txt -sjdbGTFfile Dpealeiiv2.gtf’. Optical duplicates were removed using Picard (MarkDuplicates2.18.0) (http://broadinstitute.github.io/picard/). Variants were called by mpileup and bcftools (samtools v1.6^90^) using the following parameters: samtools mpileup -A -Q 30 -d 1000 -C 50 -output-tags AD,ADF,ADR,DP,SP -uf genome.fa -b bam.md.list | bcftools call -m -A -skip-variants indels | bcftools filter -g3 -i ‘MQ>30 & SUM(DP4)>10 & (DP4[2]+DP4[3])>5’. The resulting variants were called by bcftools (samtools v1.9^90^). Variants were annotated by SnpEff v.4.3t^91^ using as reference the primary transcripts (longest isoform). Parsing of the SnpEff output was performed with customized python code *snpeff_parser_for_rnaediting.py*. Overlap of all transcriptome variants with repeats, PFAM domains, and transmembrane domains was done using bedtools intersect (bedtools v2.28.0). Edit frequencies were calculated by counting the ratio of edited sites (#G’s) over the sum of edited and not-edited sites (#A’s + #G’s). Only sites with edit frequency >0.1% were considered. For technical and biological reproducibility of our RNA editing pipeline we applied our methods to previously published dataset^10^ containing 11 transcriptomes and one genomic dataset retrieved from the Sequence Read Archive (SRA) accession record: *SRP044717*.

The fraction of Adenosines per genic feature presented in Supplementary Table 1 was by calling transcriptome variants that overlap genic regions. Sites with >10 read depth in reference call and no alternate allele were utilized to create a small vcf file and that would replace the absent alternate allele for a ‘G’ or a ‘C’ depending on the gene strand orientation. The resulting small vcf was annotated using SnpEff v.4.3t^91^ and parsed by *Parse_snpEff_nonEditedAs.py*.

#### Classification of ADAR targets

Robust edit sites are referred to those sites where at least one sample has more than 0.25 edit frequency. Classification of edit sites by tissue preference was done by analyzing the edit frequency obtained from the pooled read counts for reference and alternate transcriptome variant calls from all neural and non-neural samples, excluding retina (Ret) and axial nerve cord (ANC) as these shared weak correlations between neural and non-neural samples (Fig. 6b). The projection of Weighted Neural (WN) against Weighted Non-Neural (WNN) editing averages was used to classify the edit sites (Supplementary Note 7).

#### Protein sequence alignment for GRIK homologs

Blastp v. 2.9.0^88^ was used to identify the best scoring hits for squid proteins encoded by the genome (e value < 1e-20, -qcov_hsp_perc 0.8). MAFFT v7.245^92^ was used to align the groups of protein homologs, including the proteins resulting from RNA editing events. Alignments of homology groups are available on this link. Transmembrane domains were predicted with TMHMM v2.0^93^.

#### Genomic variant calls for cephalopod conservation in CDS regions

Genomic variants were called from sequence alignments overlapping CDS in the squid genome. Reads were aligned with bwa-mem and variant calls were made using the samtools mpileup -I -A -Q 20 | bcftools call -O z -m. High-quality genomic variant calls were required to have a minimum mapping quality of 20, and sequence depth within the expected depth for CDS regions. The expected coverage range was determined ±2 standard deviations from the mean of shotgun coverage at CDS regions for each cephalopod considered for the analysis (Supplementary Fig. 1d). Adenosines overlapping CDS in *D. pealeii* were annotated by SnpEff v.4.3t^91^. The genotype comparison between *D. opalescens*, *H. bleekeri* and *D. pealeii* were done using only sites with confident homozygous call for both *D. pealeii* specimens^10^.

### Reporting summary

Further information on research design is available in the Nature Research Reporting Summary linked to this article.

## Supplementary information

Supplementary Information

Reporting Summary

## Data Availability

The genome and transcriptome sequence reads generated in this study for *D. pealeii* are deposited as Bioproject PRJNA641326 (https://www.ncbi.nlm.nih.gov/bioproject/PRJNA641326). The genome assembly for *O. bimaculoides* generated in this study is deposited as Bioproject PRJNA808169 (http://www.ncbi.nlm.nih.gov/bioproject/808169). The *E. scolopes* sequence data used in this study is available under Bioproject PRJNA661684. (https://www.ncbi.nlm.nih.gov/bioproject/661684). The *Octopus bimaculoides* sequence data used in this study are available under Bioproject PRJNA270931 (https://www.ncbi.nlm.nih.gov/bioproject/270931). The *D. pealeii* sequenced data used in this study are available in the SRA under accession SRP044717. Source data are provided with this paper.

## Source data

Source Data

## Code availability

Code used to compute and visualize linkage group distribution, compute putative cephLGs, Dotplots/Fisher blobplots and for the RNA editing analyses is available under https://bitbucket.org/viemet/public/src/master/CephChromosomes/ (extended from^34^) and at 10.6078/D15X38.
