# Supplementary material for: Genome and transcriptome mechanisms driving cephalopod evolution
Source: Nat Commun. 2022 May 4;13:2427. doi: 10.1038/s41467-022-29748-w (PMC9068888; doi:10.1038/s41467-022-29748-w)
Supplement: Supplementary file 1 — Supplementary Information [file 41467_2022_29748_MOESM1_ESM.pdf]

Table of Contents

Supplementary Note 1: Genome assembly 3
De novo assembly of *Doryteuthis pealeii* 3
Supplementary Figure 1 5
Chromosome-scale assembly of *Octopus bimaculoides* 5
Chromosome-scale assembly of *Euprymna scolopes* 5
Supplementary Table 1. Summary of sequence libraries used in *D. pealeii* assembly 6

Supplementary Note 2: Molecular phylogeny 7
Shotgun sequencing of *Heterololigo bleekeri* 7
Shotgun sequencing of *Doryteuthis opalescens* 7
Molecular phylogeny of cephalopod lineages with mtDNA 7
Supplementary Table 2. Genbank accession numbers for Fig1b 9

Supplementary Note 3: Transcriptome sequencing 10
Supplementary Table 3. Transcriptome sequencing 11

Supplementary Note 4: Gene and repeat annotation 12
Protein-coding gene annotation 12
Gene family evolution 12
Supplementary Table 4. Clustered novel gene families (selected) 13
Repetitive Landscape 16
Supplementary Figure 2: Gene Density and repeat families 18
Supplementary Table 5. RepeatMasker report for D. pealeii. 19
Supplementary Table 6. Repeat families enriched in 100kb windows. 20

Supplementary Note 5: Chromosome Evolution 22
Mutual best-hit orthology 22
Supplementary Figure 3: Correspondence of cephalopod chromosomes 23
Supplementary Figure 4: Enrichment of orthologs of each BLG on chromosomes of six species 24
Supplementary Figure 5: Distribution of selected BLGs on cephalopod chromosomes 25
Coleoid cephalopod linkage groups (CephLGs) 25
Colinearity in cephalopod and bird genomes 25
Supplementary Table 7: Datasets used in macrosynteny analysis 26
Supplementary Table 8: Reconstruction of linkage groups 27
Supplementary Table 9. Composition of putative cephalopod linkage groups. 28
Supplementary Table 10. List of squid chromosomes that correspond 1:1 or 1:2 to octopus chromosomes. 29

Supplementary Note 6: Large Gene Families 30
Supplementary Figure 6: Large Gene Families 30

Supplementary Note 7: RNA-editing 32
Identification of confident transcriptome variants 32
Supplementary Figure 7: Editing frequency across tissues and gene elements 35
Supplementary Figure 8: Comparison of editing across tissue and editing types 36
Supplementary Figure 9: Editing in PFAM domains, transmembrane domains, and across blosum scores 37
Supplementary Figure 10: Editing profile of mRNA-associated proteins 38
Supplementary Figure 11: Editing in GRIK 38
Supplementary Figure 12: Editing profiles from genes with diverse biological function 39
Supplementary Table 11: Number of edit sites identified in the genome 40
Supplementary Table 12: Comparison of editing in previous and current study 40
Supplementary Table 13: Number of edit sites by robustness and edit type 41
Supplementary Table 14: Number of expressed adenosines in genomic features 41
Supplementary Table 15: Number of edit sites by genomic location, robustness, and repeat overlap 42
Supplementary Table 16: Identification of new and conserved robust editing sites in *D. pealeii* 43
Supplementary Table 17: New and conserved types of editing sites in *D. pealeii* 44
Supplementary Table 18: New and conserved types of robust edit sites in *D. pealeii* 45

Supplementary References 46

Supplementary Note 1: Genome assembly

De novo assembly of *Doryteuthis pealeii*

We sequenced the genome of *D. pealeii* using a whole genome shotgun approach that combined long single molecule PacBio reads with short, high accuracy paired-end Illumina data (Supplementary Table 1). Genomic DNA for all shotgun sequencing was derived from a single male individual collected in October 2015 by otter trawl from Vineyard Sound, by the Marine Resources Center at the Marine Biological Laboratory, Woods Hole, MA. The same individual was used for almost all RNA sampling, see below. All work was performed in compliance with the EU Directive 2010/63/EU on cephalopod use and AAALAC guidelines on the care and welfare of cephalopods[1–3]. For genomic DNA isolation, testis tissue was quickly dissected, flash frozen on liquid nitrogen and stored at -70°C. Genomic DNA was extracted by homogenizing and digesting testis tissue with proteinase K at 55°C overnight. A 1/3 volume of 5M NaCl was gently mixed in, and the homogenate was spun at 1000xg for 5 minutes to precipitate the protein. The supernatant was transferred to a new tube and 2 volumes of ice-cold 100% ethanol was added. High molecular weight gDNA was spooled, washed with 75% Ethanol, and resuspended overnight in nuclease-free water (Sigma) at 4°C and stored at -70°C until use. We generated a total of 1.2 billion Illumina read pairs (2x250 paired-end) and 13.4 million PacBio single molecule reads totaling 136 Gb (HudsonAlpha Institute for Biotechnology and OIST). These datasets represent 131x and 30x coverage of the 4.6 Gb *D. pealeii* genome.

We assembled the *D. pealeii* genome using a hybrid approach, aiming for a single representative haplotype across the genome. First, Illumina reads were assembled with hipmer[4] with word size k = 65 in 2D mode with gap_fill aggressive. This assembly was split into contigs at gaps and combined with all PacBio reads longer than 2.5 kb using DBG2OLC[5] (commit 1f7e752) with parameters k 17 KmerCovTh 2 MinOverlap 20 AdaptiveTh 0.005 RemoveChimera 1 MinLen 2500. The DBG2OLC contigs were then polished twice with PBDAGCON[6] (v0.3) using PacBio reads aligned by BLASR[7] (v5.3) as input (PBDAGCON params: *-t 0 -c 0*; BLASR params: *--bestn 1 --nproc 8 --minAlnLength 2500 --minPctSimilarity 70*). DBG2OLC contigs were then scaffolded with Illumina mate-pair libraries of 3.1, 6.7, and 10 kb using platanus 1.2.4[8]. After these stages the assembly totaled 4.61 Gb with contig N50 length 124.0 kb and scaffold N50 length 12.61 Mb.

To achieve a chromosome-scale assembly, we generated 136 M 2x150 paired-end HiC and 71M 2x150 and 157M 2x250 paired-end Chicago chromatin conformation capture reads (Dovetail Genomics, Scotts Valley, CA). These were assembled using HiRise[9] (Dovetail Genomics), followed by manual review and editing with the juicebox toolkit[10] (Supplementary Fig. 1). Curation revealed a common artifact of redundantly assembled sequence representing split haplotypes. We identified and removed 4235 contigs that were included entirely within larger scaffolds. Additionally, 1002 adjacent contig pairs were identified which exhibited 1 kbp or more of sequence identity > 97%. The second occurrence of the sequence was removed and

replaced with the default gap of 100 bp. The ~153 MB of sequence found redundant by these methods brought the total assembly length down from 4815 Mb to 4662 Mb of sequence.

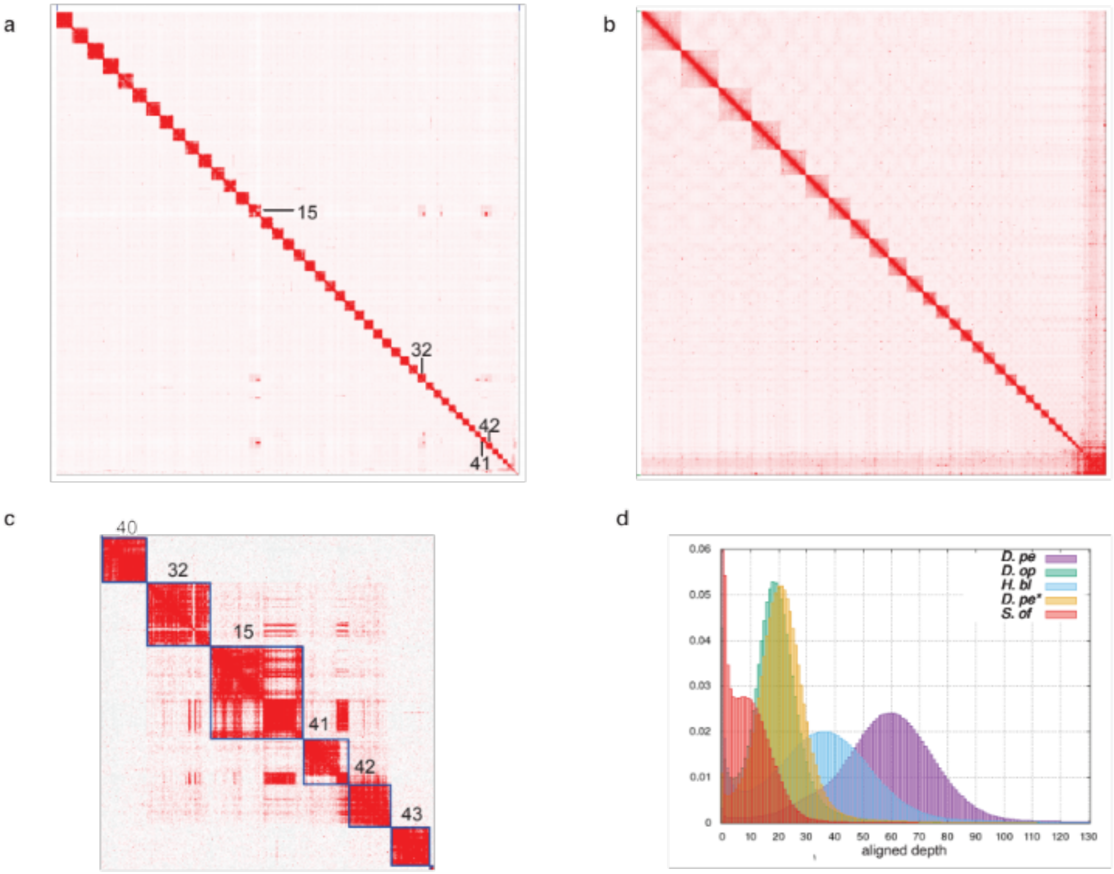

Supplementary Figure 1: Genome assembly. a: HiC contact map at mapping quality 1 of *D. pealeii* assembly. Chromosomes are ordered 1-46 from the top left corner. b: HiC contact map at mapping quality 1 of *O. bimaculoides*. Chromosomes are ordered 1-30 from the top left corner. c: A closer view of chromosomes with apparent off-diagonal signal from panel a show higher within-chromosome signal than between chromosomes. These regions are the highly repetitive C2H2/zinc finger expansions. d: aligned depth of cephalopods display single depth peaks in genic regions of the assembly.

To correct potential assembly errors we aligned long (and mate-pair) reads and used the wombat toolkit (https://gitlab.com/Bredeson/wombat) to identify regions where (1) a large proportion of the reads end at a sharp boundary and/or (2) edges of contigs at which the realigned sequencing drops off more sharply than expected. Regions without PacBio support and/or low depth were clipped. Gaps created during this process were resized using Illumina mate-pair libraries and filled using PBJelly[11]. Due to its large size, the genome could not be indexed in its entirety in the PBJelly schema. We split the problem by first partitioning reads to

their best placement on each chromosome using minimap2[12], and then running PBJelly on individual chromosomes.

Finally, the consensus sequence was polished in two passes, first with long reads using Quiver[6], and again with the Illumina short reads using a custom variant calling and patching procedure as follows. Reads were aligned using BWA-MEM[13] (v0.7.17-r1188). Variants were then called using freebayes[14] (v1.1.0-54-g49413aa; *--use-mapping-quality --genotype-qualities --report-genotype-likelihood-max --min-alternate-count 2 --min-alternate-fraction 0.05 --min-base-quality 20 --min-mapping-quality 20 --min-repeat-entropy 1 --max-complex-gap -1 --strict-vcf*) and homozygous variants (SNVs and indels) within the depth range 19-210 were selected. These variants were used to correct the sequence of the assembly using a custom Python script called ILEC (v0.1.3; https://bitbucket.org/rokhsar-lab/map4cns).

The final *D. pealeii* assembly spans 4.595 Gb, with 94.4% of base pairs in N=46 chromosomes, consistent with the loliginid karyotype[15,16], and the remainder in smaller scaffolds (<636 kb) that could not be confidently placed on chromosomes. Half of the assembled base pairs are in contigs longer than 230.8 kb.

To estimate the heterozygosity of the reference individual we aligned Illumina paired-end reads to the final genome assembly and called single nucleotide variants with freebayes[14]. To avoid overestimating the variant rate, we considered only sites that were confidently covered at the expected diploid rate, which was operationally defined as within two standard deviations of the modal depth.

Chromosome-scale assembly of *Octopus bimaculoides*

To produce a chromosome-scale assembly for *O. bimaculoides*, we integrated new HiC datasets (Bioproject PRJNA808169 http://www.ncbi.nlm.nih.gov/bioproject/808169) with the previously published shotgun assembly[17]. Since the aim was to produce a substrate for chromosome-scale analyses of synteny, we did not attempt to improve upon the shotgun assembly or close any gaps. Initial scaffolding was done with HiRise[9] and curated with juicebox tools[10]. We recovered 2.37 Gb in N=30 chromosomes with 0.14 Gb in smaller scaffolds that could not be confidently placed on chromosomes. Commensurately, 94.2% of previously annotated genes are now placed on chromosomes.

Chromosome-scale assembly of *Euprymna scolopes*

The *Euprymna scolopes* assembly (PRJNA645380) was generated using previously obtained HiC data and assembled with Lachesis[18,19]. Briefly, scaffolds of 50kb and longer from the publicly available[20] assembly were used together with aligned Hi-C reads. We used HiCPro[21] with the following settings:
--very-sensitive -L 30 --score-min L,-0.6,-0.2 --end-to-end --reorder. Local options= --very-sensitive -L 20 --score-min L,-0.6,-0.2 --end-to-end –reorder Normalization maxiter = 100 filterlowcountpercent = 0.02 filterhighcountpercent = 0 EPS = 0.1. Different chromosome

numbers were tested for clustering until the cleanest heatmap result was obtained in Lachesis. Using *CLUSTER_N 46* we assembled the genome into 48 chromosomes. Other Lachesis parameters were: *RE_SITE_SEQ AAGCTT, CLUSTER_CONTIGS_WITH_CENS = -1, CLUSTER_MIN_RE_SITES = 25, CLUSTER_MAX_LINK_DENSITY = 2, CLUSTER_NONINFORMATIVE_RATIO = 3, CLUSTER_DRAW_HEATMAP = 0, CLUSTER_DRAW_DOTPLOT = 1, ORDER_MIN_N_RES_IN_TRUNK = 15, ORDER_MIN_N_RES_IN_SHREDS = 15*).

Supplementary Table 1. Summary of sequence libraries used in *D. pealeii* assembly

**Paired-end sequence**			
**Library name**	**Sequence type**	**Reads (million)**	**Total length (Gbp)**
IERA	Illumina 2x250, 400 bp frag	532.14	266.07
IERB	Illumina 2x250, 400 bp frag	373.66	186.83
IERC	Illumina 2x250, 800 bp frag	668.72	334.36
IERD	Illumina 2x250, 800 bp frag	827.94	413.97
Total paired-end		2,402.46	1201.23
**Mate-pair sequence**			
**Library name**	**Sequence type**	**Reads (million)**	**Total length (Gbp)**
IERE	2.8 KB mate-pair	82.46	12.5
IERF	6 KB mate-pair	113.68	17.2
IERG	6 KB mate-pair	133.24	20.2
IERH	9.3 KB mate-pair	70.28	10.7
IEXF	2.3 KB mate-pair	118.32	17.9
**PacBio long-read sequence**			
**Library name**		**Reads**	**Total length (Gbp)**
HudsonAlpha		8,132,524	78.22
RokU_72		302,913	2.98
RokU_74		219,089	2.43
RokU_76		354,794	3.87
RokU_77		322,961	3.72
RokU_78		923,979	9.78
RokU_79		762,636	7.99
RokU_80		874,043	9.94
RokU_81		1,010,229	11.56
RokU_82		471,442	5.20
Total PacBio		13,374,610	135.71

Supplementary Note 2: Molecular phylogeny

Shotgun sequencing of *Heterololigo bleekeri*

To obtain genomic data from a divergent loliginid, we collected a fresh individual of *H. bleekeri* from a fishing port in Hakodate city, Hokkaido Prefecture (Japan), in November 2016. The spermatophores of this individual were removed and submerged in RNAlater (Qiagen) for at least two hours at room temperature before storing at -80 degrees. High molecular weight genomic DNA was isolated from the spermatophores using the nuclei DNA isolation described in[22]. We used the Illumina TruSeq DNA PCR-Free LT Library Prep Kit (Illumina, San Diego, CA), with an insert size of 300 bp to construct a genomic library. The library was sequenced with 2x150 paired-end reads using two lanes of the HiSeq 4000 Illumina platform at the OIST Sequencing Center.

Shotgun sequencing of *Doryteuthis opalescens*

Adult *Doryteuthis opalescens* were caught by jig off the Hopkins Marine Station in Monterey, CA. All work was performed in compliance with the EU Directive 2010/63/EU on cephalopod use and AAALAC guidelines on the care and welfare of cephalopods[1–3]. Specimens were transported back to the lab and anesthetized with 2% ethanol in seawater and euthanized by decapitation followed by transecting the brain. Testis tissue was isolated and flash frozen on liquid nitrogen and stored at -70°C. Genomic DNA was extracted by homogenizing and digesting testis tissue with proteinase K at 55 degrees overnight. A 1/3 volume of 5M NaCl was mixed in, and the homogenate was spun at 1000xg for 5 minutes to precipitate the protein. The supernatant was transferred to a new tube and 2 volumes of ice-cold 100% ethanol was added. High molecular weight gDNA was spooled, washed with 75% Ethanol, and resuspended overnight in nuclease-free water (Sigma) at 4°C, and stored at -70°C until use. Genomic DNA was sequenced using 2x100 and 2x150 paired-end reads at the Functional Genomics Facility at the University of Chicago.

Molecular phylogeny of cephalopod lineages with mtDNA

We inferred molecular phylogeny and divergence times of nineteen species that comprise the major cephalopod lineages using mitochondrial protein-coding genes. We selected seventeen species that have their full mitochondrial genome available in Genbank and as representatives of unique clades (Supplementary Table 2), notably those deposited by Almeida et al.[23] We assembled the mitochondrial genomes of *D. pealeii* and *H. bleekeri* from whole genome shotgun data sequenced in this study using NOVOplasty 4.0[24]. For *O. bimaculoides* we assembled the mitochondrial contig *de novo* from previously generated whole genome shotgun reads[17] with NOVOplasty[24] using the *Octopus vulgaris* mitochondrial genome as a seed. For *Loligo vulgaris* and *E. scolopes* no mtDNA sequence was available, and we assembled transcriptome data (Supplementary Table 2) *de novo* using Trinity[25] and extracted mitochondrial protein-coding transcripts.

For each species, we retrieved open reading frames using the standalone ORFfinder (https://www.ncbi.nlm.nih.gov/orffinder/). The mitochondrial protein-coding genes were annotated based on the best hit between amino-acid sequences of our target species with the mitochondrial protein-coding genes of *Idiosepius sp.* (accession number KF647895) after blastp in the standalone NCBI BLAST v2.10 (https://ftp.ncbi.nlm.nih.gov/blast/executables/blast+/LATEST/).

We performed maximum likelihood analyses of concatenated sequences in IQ-TREE[26] with the best model and partition scheme selected by ModelFinder [27], and 1000 replicates of ultrafast likelihood bootstrap[28]. Before concatenation, each protein-coding gene was aligned codon-based using MUSCLE 3.8[29] implemented in AliView[30].

We estimated the age of each node assuming a strict clock with the Langley–Fitch method in r8s 1.8[31]. For these estimations, we rooted the tree generated in IQ-TREE and fixed the age of two internal nodes, one for the crown Coleoidea to 328 and 254 Mya, and the other for the divergence between the Vampyromorphida and Octobrachia to 276 and 206 Mya. These ages correspond to the maximum and minimum age estimations using transcriptome data from Supplementary Figure 4 in[32].

The chronogram shown in Fig. 1b shows both the minimum and maximum fixed age chronograms in r8s. For most the nodes, our divergence estimations fall within or were close to previously reported estimations using transcriptome data from all major cephalopod lineages in [32], and large ribosomal subunit and rhodopsin from loliginid squids (Loliginidae) in [33] despite the different source of calibration. For practical reasons, we show our estimated chronogram without the outgroup *Nautilus macromphalus*.

Supplementary Table 2. Genbank accession number of additional species used for the divergence time estimations. * Genome reads; ** Transcriptome reads.

Species	Accession Number
Nautilus macromphalus	NC_007980
Vampyroteuthis infernalis	NC_009689
Amphioctopus fangsiao	NC_007896
Octopus minor	HQ638215
Octopus bimaculoides	SRR2005790*
Octopus vulgaris	NC_006353
Sepia esculenta	NC_009690
Sepia officinalis	NC_007895
Loligo vulgaris	SRR3472303**
Sepioteuthis lessoniana	NC_007894
Doryteuthis opalescens	NC_012840
Euprymna scolopes	SRR8172560**
Bathyteuthis abyssicola	AP012225
Architeuthis dux	FJ429092
Todarodes pacificus	NC_006354
Dosidicus gigas	NC_009734
Sthenoteuthis oualaniensis	EU660577

Supplementary Note 3: Transcriptome sequencing

We generated transcriptomes from twenty-eight different tissues to aid gene prediction and to enable expression and RNA editing analyses (Supplementary Table 3). All tissues except for the blood and posterior salivary gland (PSG) sample were obtained from the same adult male specimen that provided genomic DNA for shotgun sequencing. This allowed us to confidently distinguish A-to-I editing sites from genomic heterozygosity (see below). We chose tissues that cover a wide range of anatomical and neurological structures with an eye towards identifying and characterizing genes expressed in structures that feature cephalopod innovations. All tissues harvested were quickly dissected and flash frozen on liquid nitrogen with a small amount of Trizol. As a control for RNA degradation, we sampled one optic lobe at the start of dissection and the other at the end, separated by approximately 30 minutes. These are designated OL-early and OL-late, and analysis showed that there was little difference in gene expression and A-to-I editing between these two samples. Skin samples were isolated from dorsal and ventral mantle as well as the dorsal fin. Dorsal and ventral mantle tissues were carefully separated into the chromatophore and iridophore layers before flash freezing in liquid nitrogen. Samples were stored at -70°C. RNA was isolated using Trizol (Invitrogen) following manufacturer's instructions.

RNA integrity was analyzed with a Bioanalyzer 2100; only samples with clean rRNA peaks and little to no degradation were used. Total RNA was polyA selected and directionally sequenced at the University of Chicago Genomics Facility on an Illumina HiSeq2000 per manufacturer's instructions, generating paired-end 2 x 100 bp reads with an insert size of ~300 bp. These reads are deposited in Bioproject PRJNA641326.

Supplementary Table 3. Transcriptome sequencing obtained from two squid specimens.

	ID	Tissue	Specimen	trimmed reads	% STAR unique	% STAR aligned
1	VL	Vertical_lobe	A	138,120,630	82.60%	93.18%
2	Sup	Supraesophageal_brain	A	135,770,518	65.08%	91.22%
3	Sub	Subesophageal_brain	A	141,414,112	75.97%	94.42%
4	OLe	Optic_Lobe_early	A	134,643,662	81.55%	94.72%
5	OLl	Optic_Lobe_late	A	133,594,698	81.00%	94.29%
6	GFL	Giant_Fiber_Lobe	A	133,763,432	73.63%	95.35%
7	SG	Stellate_Ganglion	A	131,810,100	73.11%	94.83%
8	BuL	Buccal_Lobe	A	133,271,884	67.89%	92.51%
9	BrL	Brachial_Lobe	A	133,687,352	82.38%	94.20%
10	ANC	Axial_Nerve_Cord	A	139,472,068	87.89%	96.07%
11	Tes	Testes	A	138,319,052	84.81%	92.43%
12	Ten	Tentacles	A	138,591,744	86.13%	94.35%
13	Suc	Suckers	A	145,216,842	86.85%	94.27%
14	Ret	Retina	A	135,376,682	82.25%	90.47%
15	Man	Mantle	A	158,268,776	88.20%	96.35%
16	Kid	Kidney	A	158,718,910	78.42%	94.48%
17	Ink	Ink	A	148,008,024	6.95%	83.66%
18	Hep	Hepatopancreas	A	151,400,392	82.44%	93.46%
19	Hea	Heart	A	127,714,976	75.88%	93.61%
20	Gil	Gill	A	131,400,130	81.54%	94.85%
21	Blo	Blood	B	136,112,846	84.32%	90.94%
22	BM	Buccal_Mass	A	136,081,906	79.21%	93.29%
23	DCI	Skin_FDCI_-_ Dorsal_Fin_Chromatophore_Iridophore_layers	A	130,058,454	83.98%	91.96%
24	MDC	Skin_MDC_-_ Dorsal_Mantle_Chromatophore_layer	A	135,439,046	85.86%	93.80%
25	MDI	Skin_MDI_-_ Dorsal_Mantle_Iridophore_layer	A	131,220,862	82.56%	90.95%
26	MVC	Skin_MVC_-_ Ventral_Mantle_Chromatophore_layer	A	133,728,940	88.09%	94.44%
27	MVI	Skin_MVI_Ventral_Mantle_Iridophore_layer	A	130,850,842	77.33%	87.89%
28	PSG	Posterior_Salivary_Gland	B	134,249,418	86.77%	92.37%

Supplementary Note 4: Gene and repeat annotation

Protein-coding gene annotation

We annotated protein-coding genes of the *D. pealeii* genome using the DOE Joint Genome Institute (JGI) annotation pipeline (img.jgi.doe.gov/docs/pipelineV5/) which uses transcriptional evidence, homology support, and *ab initio* methods to predict and confirm protein coding genes. RNA-seq data from 27 tissues (PRJNA641326) were aligned to the genome and assembled on-genome into transcripts by PERTRAN[34]. Assembled transcripts were aligned to the genome using PASA[35], and PASA alignments, along with exonerate alignments of the proteomes of *Octopus bimaculoides, Aplysia californica, Crassostrea virginica, Homo sapiens, Xenopus tropicalis, Lottia gigantea,* and Swissprot eukaryotes (downloaded November 2017). The alignments and peptide homology sequences of the transcript assemblies and the peptides were submitted to GenomeScan[36] and Fgenesh+[37] for gene predictions. A best prediction per locus was selected and used to add UTR, to correct intron/exon boundaries with transcript data, and to add additional splice isoforms with PASA. Genes were then filtered by requiring protein homology, domain annotation, or some transcriptional evidence for inclusion in the final gene set.

In total, we predicted the structure of 24,911 protein-coding genes. Using BUSCO[38] to assess assembly completeness and annotation quality, we find that 92.4% of the core Eukaryota (v9) genes are complete, and an additional 4% are present but fragmented. 5.3% were marked by BUSCO as duplicated. 18,296 of these have a Swiss-Prot homolog (blastp e-value<=0.001). Similarly, 18,774 *Euprymna scolopes* genes and 17,198 *Octopus bimaculoides* genes have Swiss-Prot homologs, suggesting the coleoid gene number is largely conserved.

Gene family evolution

Gene families of particular interest were manually curated and analyzed as described in [17]. Briefly, we searched for candidate genes in the *D. pealeii* genome and transcriptome assemblies, as well as the proteomes of *Homo sapiens*, *Mus musculus, Drosophila melanogaster, Tribolium castaneum, Caenorhabditis elegans, Capitella teleta, Crassostrea gigas, Lottia gigantea,* and *Octopus bimaculoides,* using BLASTP and TBLASTN. Candidate genes were verified using BLAST and Pfam. Genes identified in the *D. pealeii* genome were confirmed and extended using the transcriptomes, and multiple gene models that matched the same transcript were combined. The identified sequences from octopus and other bilaterians were aligned using either MUSCLE[29] or CLUSTALO[39]. Phylogenetic trees were constructed with FastTree2[40] using full-length sequences, and visualized with Figtree (A. Rambaut, http://tree.bio.ed.ac.uk/software/figtree/).

Supplementary Table 4. Clustered novel gene families (selected) identified by reciprocol BLASTP of individual chromosomes.

GeneID	Chromosome	Notes	Expression
Dopeav2001628m	Dpe01		multiple tissues
Dopeav2001629m	Dpe01		multiple tissues
Dopeav2001631m	Dpe01		multiple tissues
Dopeav2001632m	Dpe01		multiple tissues
Dopeav2001633m	Dpe01		multiple tissues
Dopeav2001634m	Dpe01		multiple tissues
Dopeav2001635m	Dpe01		multiple tissues
Dopeav2001636m	Dpe01		multiple tissues
Dopeav2001637m	Dpe01		multiple tissues
Dopeav2001638m	Dpe01		multiple tissues
Dopeav2001639m	Dpe01		multiple tissues
Dopeav2001642m	Dpe01		multiple tissues
Dopeav2001643m	Dpe01		multiple tissues
Dopeav2001645m	Dpe01		multiple tissues
Dopeav2001646m	Dpe01		multiple tissues
Dopeav2001647m	Dpe01		multiple tissues
Dopeav2001654m	Dpe01		multiple tissues
Dopeav2001655m	Dpe01		multiple tissues
Dopeav2138511m	Dpe01		multiple tissues
Dopeav2006500m	Dpe02	IL17	dorsal fin skin
Dopeav2006502m	Dpe02	IL17	dorsal fin skin
Dopeav2006503m	Dpe02	IL17	dorsal fin skin
Dopeav2006504m	Dpe02	IL17	dorsal fin skin
Dopeav2006505m	Dpe02	IL17	dorsal fin skin
Dopeav2006506m	Dpe02	IL17	dorsal fin skin

Dopeav2006507m	Dpe02	IL17	dorsal fin skin
Dopeav2006509m	Dpe02	IL17	dorsal fin skin
Dopeav2006510m	Dpe02	IL17	dorsal fin skin
Dopeav2006511m	Dpe02	IL17	dorsal fin skin
Dopeav2006513m	Dpe02	IL17	dorsal fin skin
Dopeav2006514m	Dpe02	IL17	dorsal fin skin
Dopeav2006516m	Dpe02	IL17	dorsal fin skin
Dopeav2006517m	Dpe02	IL17	dorsal fin skin
Dopeav2006519m	Dpe02	IL17	dorsal fin skin
Dopeav2006520m	Dpe02	IL17	dorsal fin skin
Dopeav2006522m	Dpe02	IL17	dorsal fin skin
Dopeav2006523m	Dpe02	IL17	dorsal fin skin
Dopeav2006524m	Dpe02	IL17	dorsal fin skin
Dopeav2006525m	Dpe02	IL17	dorsal fin skin
Dopeav2006526m	Dpe02	IL17	dorsal fin skin
Dopeav2006529m	Dpe02	IL17	dorsal fin skin
Dopeav2006531m	Dpe02	IL17	dorsal fin skin
Dopeav2006532m	Dpe02	IL17	dorsal fin skin
Dopeav2006533m	Dpe02	IL17	dorsal fin skin
Dopeav2006534m	Dpe02	IL17	dorsal fin skin
Dopeav2006535m	Dpe02	IL17	dorsal fin skin
Dopeav2006536m	Dpe02	IL17	dorsal fin skin
Dopeav2138482m	Dpe02	IL17	dorsal fin skin
Dopeav2006722m	Dpe02	suckerins	tentacle
Dopeav2006723m	Dpe02	suckerins	tentacle
Dopeav2006726m	Dpe02	suckerins	tentacle
Dopeav2006727m	Dpe02	suckerins	tentacle
Dopeav2006730m	Dpe02	suckerins	tentacle
Dopeav2006732m	Dpe02	suckerins	tentacle

Dopeav2006734m	Dpe02	suckerins	tentacle
Dopeav2006736m	Dpe02	suckerins	tentacle
Dopeav2006738m	Dpe02	suckerins	tentacle
Dopeav2006741m	Dpe02	suckerins	tentacle
Dopeav2006800m	Dpe02	suckerins	tentacle
Dopeav2006801m	Dpe02	suckerins	tentacle
Dopeav2006803m	Dpe02	suckerins	tentacle
Dopeav2027120	Dpe07	Chitin-binding	buccal mass
Dopeav2027121	Dpe07	Chitin-binding	buccal mass
Dopeav2027122	Dpe07	Chitin-binding	buccal mass
Dopeav2027125	Dpe07	Chitin-binding	buccal mass
Dopeav2027126	Dpe07	Chitin-binding	buccal mass
Dopeav2027127	Dpe07	Chitin-binding	buccal mass
Dopeav2027129	Dpe07	Chitin-binding	buccal mass
Dopeav2027130	Dpe07	Chitin-binding	buccal mass
Dopeav2027132	Dpe07	Chitin-binding	buccal mass
Dopeav2027133	Dpe07	Chitin-binding	buccal mass
Dopeav2027136	Dpe07	Chitin-binding	buccal mass
Dopeav2005254m	Dpe10		tentacle
Dopeav2005255m	Dpe10		tentacle
Dopeav2037098m	Dpe10		skin
Dopeav2037099m	Dpe10		skin
Dopeav2037103m	Dpe10		skin
Dopeav2037104m	Dpe10		skin
Dopeav2037105m	Dpe10		skin
Dopeav2037107m	Dpe10		skin
Dopeav2041977m	Dpe12	Histidine-rich buccal mass proteins	buccal mass

Dopeav2041978m	Dpe12	Histidine-rich buccal mass proteins	buccal mass
Dopeav2041980m	Dpe12	Histidine-rich buccal mass proteins	buccal mass
Dopeav2041983m	Dpe12	Histidine-rich buccal mass proteins	buccal mass
Dopeav2041984m	Dpe12	Histidine-rich buccal mass proteins	buccal mass
Dopeav2041985m	Dpe12	Histidine-rich buccal mass proteins	buccal mass
Dopeav2041986m	Dpe12	Histidine-rich buccal mass proteins	buccal mass
Dopeav2041987m	Dpe12	Histidine-rich buccal mass proteins	buccal mass
Dopeav2041989m	Dpe12	Histidine-rich buccal mass proteins	buccal mass
Dopeav2082845m	Dpe24		hepatopancreas
Dopeav2082849m	Dpe24		hepatopancreas
Dopeav2082854m	Dpe24		hepatopancreas
Dopeav2082983m	Dpe24		hepatopancreas
Dopeav2083211m	Dpe24		hepatopancreas
Dopeav2083218m	Dpe24		hepatopancreas

Repetitive Landscape

Repeats were annotated using the commonly used automated pipeline RepeatModeler (2.0)[41] and RepeatMasker (open-4.0.7)[42]. RepeatMasker was run with NCBI/RMBLAST (2.6.0+) and RepeatMasker library dc20170127-rb20170127. Repeatmodeler libraries contained 2,336 elements for *Doryteuthis pealeii*, 2,132 for *Octopus bimaculoides,* and 1,603 for *Euprymna scolopes*. Overall masking levels can vary from 46% (*O. bimaculoides*) to 62% (*D. pealeii*) also due to different sequencing and assembly strategies (Illumina only for *O. bimaculoides*, including gaps which are likely repeats). Overall mean GC content was 33.76%.

Ks estimation using yn00 (PAML package V4.9e,[43]) on protein directed CDS alignments were run on mutual best hits between two species pairs (*D. pealeii – D. opalescens, D. pealeii – H. bleekerei, D. pealeii – E. scolopes and D. opalescens – H. bleekeri*). This resulted in median distances of 0.0375 for *D. opalescens*, 0.1355 for *H. bleekeri* and 1.2094 for *E. scolopes*. Comparing TE distance and Ks estimation could indicate that 10% (0.1 substitutions per site) might constitute mostly genus specific expansions.

We find that the *D. pealeii* genome is 61% repetitive. As with genes, repetitive elements are also unevenly distributed across the genome. We found 40 repeat families (of different classes, out of 2,357 total repeat families) that are concentrated in a single 100 kb window per chromosome

on at least 5 chromosomes (Supplementary Table 6). None of these elements, however, occur across chromosomes in squid or octopus (median of 6 chromosomes), suggesting that different chromosomes have supported the expansion of distinct localized repeat classes.

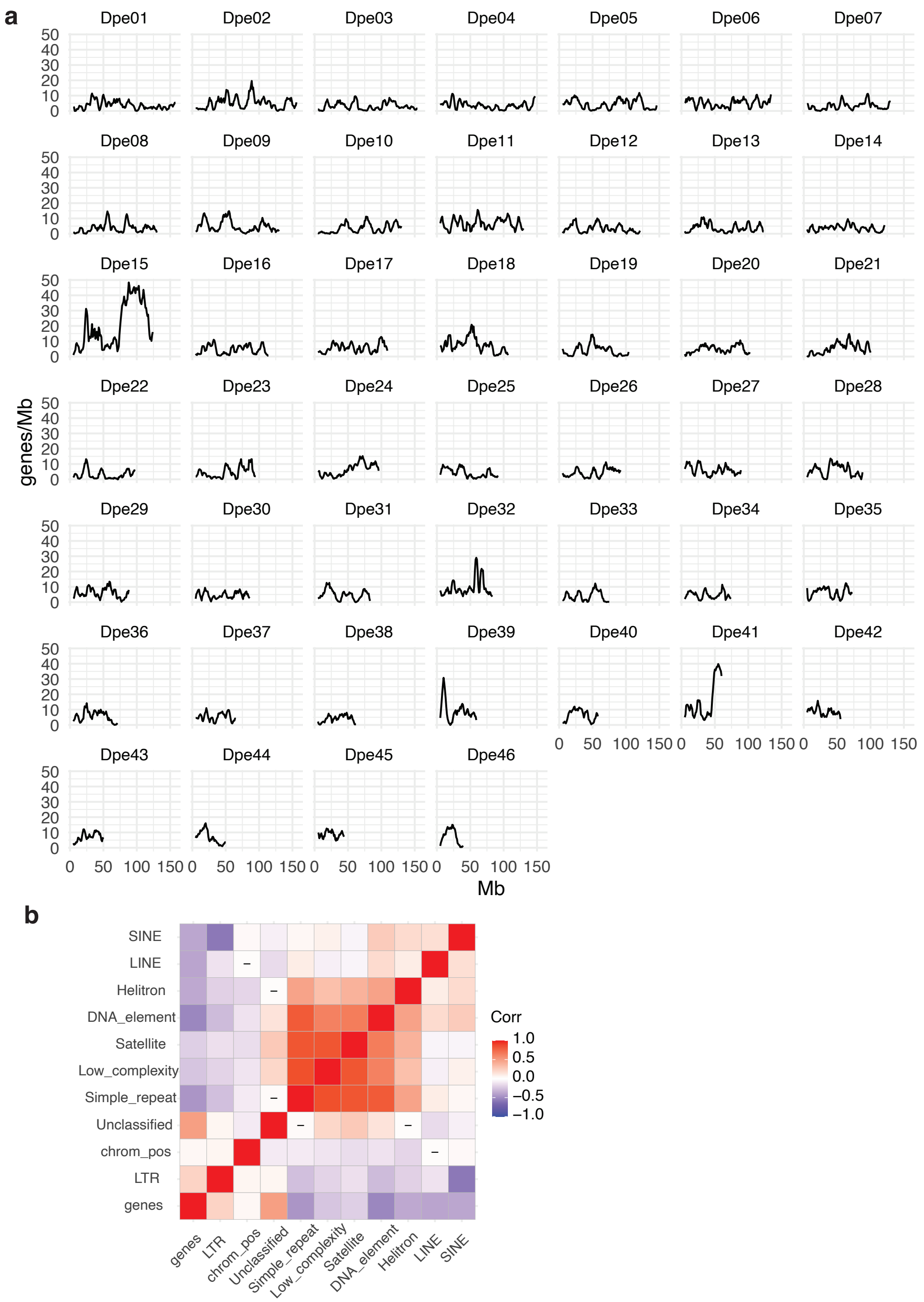

Supplementary Figure 2. a: Gene density in 5 Mbp windows for each *D. pealeii* chromosome. b: The correlation of repetitive element classes, genes, and chromosomal positions. The repeat density, gene count, and normalized position relative to the nearest chromosome end (chrom_pos) was calculated in 5 MB bins and the Pearson correlation coefficient was calculated. Comparisons marked with “-” showed no significant correlation (p<=0.05, two-sided, no correction for multiple tests). Source data are provided as a Source Data file.

Supplementary Table 5. RepeatMasker report (* most repeats fragmented by insertions or deletions have been counted as one element) for *D. pealeii.*

Type	Sub-type	Number of elements*	Length of genome covered (base pairs)	Fraction of genome sequence
SINEs		1,851,243	278,882,616	6.07%
	ALUs	0	0	0.00%
	MIRs	0	0	0.00%
LINEs		2,997,830	664,575,849	14.45%
	LINE1	3,725	662,464	0.01%
	LINE2	29,903	6,837,499	0.15%
	L3/CR1	35,691	8,621,159	0.19%
LTR elements		19,462	41,160,800	0.90%
	ERVL	0	0	0.00%
	ERVL-MaLRs	0	0	0.00%
	ERV_class I	586	115,202	0.00%
	ERV_class II	121,329	18,172,987	0.40%
DNA elements		2,058,777	394,928,791	8.59%
	hAT-Charlie	55,519	11,257,083	0.24%
	TcMar-Tigger	58,496	10,685,192	0.23%
Unclassified		5,433,091	1,060,507,626	23.06%
Total interspersed repeats			2,440,055,682	53.07%
Small RNA:		1,605,572	249,007,756	5.42%
Satellites:		141,478	19,093,006	0.42%
Simple repeats:		5,764,188	350,790,350	7.63%
Low complexity:		481,077	61,183,052	1.33%

Supplementary Table 6. Repeat families enriched in 100kb windows.

Repeatmodeler family	No. chr	Chromosomes (counts per 100kb)
rnd-1_family-455	6	Dpe36 (70), Dpe19 (12), Dpe39 (27), Dpe26 (22), Dpe28 (48), Dpe27 (15)
rnd-1_family-393	6	Dpe05 (30), Dpe15 (55), Dpe36 (31), Dpe37 (26), Dpe40 (66), Dpe34 (29)
rnd-1_family-754	8	Dpe07 (16), Dpe35 (19), Dpe19 (27), Dpe34 (23), Dpe23 (31), Dpe25 (56), Dpe28 (25), Dpe20 (71)
rnd-1_family-795	15	Dpe30 (38), Dpe33 (16), Dpe15 (32), Dpe11 (62), Dpe04 (93), Dpe21 (22), Dpe26 (26), Dpe05 (68), Dpe42 (28), Dpe16 (24), Dpe12 (50), Dpe24 (17), Dpe27 (20), Dpe23 (61), Dpe32 (14)
rnd-1_family-531	5	Dpe07 (27), Dpe29 (34), Dpe14 (16), Dpe22 (15), Dpe30 (20)
rnd-1_family-1039	5	Dpe06 (11), Dpe38 (34), Dpe44 (71), Dpe37 (16), Dpe05 (20)
rnd-1_family-367	8	Dpe44 (55), Dpe29 (125), Dpe03 (24), Dpe36 (33), Dpe39 (20), Dpe42 (49), Dpe38 (60), Dpe19 (38)
rnd-1_family-409	6	Dpe03 (78), Dpe23 (55), Dpe31 (49), Dpe14 (80), Dpe45 (99), Dpe25 (249)
rnd-6_family-1435	5	Dpe13 (13), Dpe16 (27), Dpe41 (24), Dpe42 (34), Dpe14 (22)
rnd-1_family-1234	16	Dpe22 (21), Dpe15 (55), Dpe02 (28), Dpe45 (16), Dpe28 (26), Dpe26 (74), Dpe03 (43), Dpe33 (23), Dpe41 (87), Dpe43 (17), Dpe34 (31), Dpe06 (16), Dpe13 (12), Dpe17 (22), Dpe40 (24), Dpe29 (16)
rnd-1_family-751	6	Dpe21 (54), Dpe04 (23), Dpe19 (21), Dpe01 (12), Dpe22 (16), Dpe16 (18)
rnd-1_family-449	7	Dpe37 (26), Dpe30 (15), Dpe10 (24), Dpe09 (32), Dpe45 (26), Dpe36 (120), Dpe25 (136)
rnd-4_family-174	5	Dpe15 (31), Dpe40 (19), Dpe08 (33), Dpe38 (14), Dpe17 (14)
rnd-1_family-1225	13	Dpe29 (17), Dpe34 (20), Dpe26 (71), Dpe43 (16), Dpe28 (25), Dpe33 (23), Dpe03 (43), Dpe41 (25), Dpe06 (18), Dpe15 (57), Dpe40 (23), Dpe42 (22), Dpe22 (19)
rnd-1_family-1111	6	Dpe32 (24), Dpe45 (17), Dpe29 (53), Dpe31 (31), Dpe40 (12), Dpe28 (35)
rnd-1_family-900	5	Dpe16 (46), Dpe29 (25), Dpe33 (61), Dpe35 (62), Dpe22 (34)
rnd-1_family-45	6	Dpe10 (33), Dpe22 (49), Dpe02 (36), Dpe19 (52), Dpe32 (61), Dpe14 (32)
rnd-5_family-1117	5	Dpe43 (15), Dpe37 (33), Dpe25 (16), Dpe20 (14), Dpe10 (29)
rnd-1_family-919	5	Dpe37 (18), Dpe11 (20), Dpe25 (47), Dpe35 (22), Dpe34 (10)
rnd-1_family-362	5	Dpe17 (129), Dpe21 (16), Dpe05 (68), Dpe18 (103), Dpe30 (46)
rnd-1_family-417	5	Dpe18 (83), Dpe29 (34), Dpe41 (15), Dpe37 (55), Dpe19 (53)

rnd-1_family-493	8	Dpe41 (20), Dpe16 (63), Dpe29 (62), Dpe03 (19), Dpe25 (26), Dpe32 (35), Dpe12 (34), Dpe42 (18)
rnd-1_family-276	7	Dpe13 (28), Dpe44 (35), Dpe09 (28), Dpe19 (10), Dpe15 (81), Dpe45 (29), Dpe43 (109)
rnd-6_family-102	12	Dpe40 (24), Dpe43 (14), Dpe34 (25), Dpe42 (23), Dpe06 (16), Dpe29 (15), Dpe17 (19), Dpe03 (43), Dpe02 (28), Dpe45 (16), Dpe26 (70), Dpe22 (21)
rnd-1_family-1094	7	Dpe29 (34), Dpe38 (35), Dpe04 (15), Dpe08 (220), Dpe18 (37), Dpe41 (10), Dpe10 (15)
rnd-4_family-3195	7	Dpe01 (31), Dpe33 (26), Dpe20 (51), Dpe46 (42), Dpe42 (22), Dpe39 (128), Dpe40 (366)
rnd-1_family-507	6	Dpe28 (58), Dpe02 (20), Dpe42 (17), Dpe27 (41), Dpe01 (44), Dpe37 (21)
rnd-1_family-838	6	Dpe34 (29), Dpe41 (26), Dpe24 (61), Dpe22 (31), Dpe09 (78), Dpe02 (17)
rnd-1_family-910	7	Dpe40 (28), Dpe29 (37), Dpe30 (40), Dpe28 (108), Dpe10 (26), Dpe21 (22), Dpe36 (37)
rnd-5_family-463	6	Dpe19 (24), Dpe03 (25), Dpe26 (98), Dpe05 (23), Dpe08 (28), Dpe39 (45)
rnd-1_family-350	9	Dpe02 (14), Dpe34 (57), Dpe15 (28), Dpe25 (18), Dpe36 (16), Dpe39 (14), Dpe23 (17), Dpe41 (57), Dpe16 (16)
rnd-4_family-1730	6	Dpe30 (47), Dpe33 (29), Dpe22 (19), Dpe21 (29), Dpe05 (143), Dpe42 (118)
rnd-4_family-269	6	Dpe38 (36), Dpe05 (89), Dpe33 (40), Dpe01 (52), Dpe46 (29), Dpe43 (140)
rnd-1_family-826	7	Dpe01 (36), Dpe35 (17), Dpe36 (40), Dpe29 (10), Dpe41 (40), Dpe44 (10), Dpe06 (12)
rnd-1_family-124	7	Dpe32 (40), Dpe12 (72), Dpe41 (36), Dpe43 (24), Dpe40 (78), Dpe15 (26), Dpe20 (16)
rnd-1_family-43	6	Dpe45 (27), Dpe09 (77), Dpe32 (20), Dpe39 (58), Dpe28 (28), Dpe15 (35)
rnd-6_family-3259	6	Dpe07 (15), Dpe23 (78), Dpe22 (29), Dpe28 (41), Dpe30 (52), Dpe34 (12)
rnd-1_family-611	6	Dpe25 (23), Dpe39 (24), Dpe26 (36), Dpe30 (42), Dpe22 (21), Dpe43 (25)
rnd-1_family-351	6	Dpe38 (19), Dpe04 (14), Dpe29 (29), Dpe40 (40), Dpe43 (20), Dpe08 (13)
rnd-1_family-524	5	Dpe40 (56), Dpe06 (94), Dpe41 (13), Dpe36 (42), Dpe43 (65)

Supplementary Note 5: Chromosome Evolution

Mutual best-hit orthology

Reciprocal BLASTP (version 2.10.0+,[44]) for *E. scolopes, D. pealeii O. bimaculoides,* and *M. yessoensis* was run against *B. floridae.* Mutual best hits (MBHs, e-value cutoff 1e-2) were combined to form 6,821 core orthologs. Orthologs of this core set were then identified in the remaining species (Supplementary Table 7), by computing their Mutual Best Hits to *B. floridae* and merging with the 6,821 gene core set. The orthology information of cephalopod genes enabled the reconstruction of chromosomal dynamics within cephalopods. Orthologous gene content on the 46 *D. pealeii* chromosomes and 46 *E. scolopes* chromosomal scaffolds shows that there is almost a 1:1 correspondence between decapodiform chromosomes (using at least 10 orthologous genes per Bilaterian Linkage Group (BLG)), while most octopus chromosomes correspond to several squid chromosomes (Supplementary Fig. 3a). We removed 2 additional potential chromosomal scaffolds of *E. scolopes* that were much smaller than other chromosomes (1604139bp and 1393269bp in comparison to 9560946bp of the next largest scaffold) and showed little orthology. Corresponding chromosomes are significantly enriched in shared orthologues (Bonferroni multiple test corrected, using all shared core orthologues between two species)(Supplementary Fig. 3b).

A custom script was used to extract the genomic locations of those orthologs from their genome annotations. Shared orthologs between species were clustered with Euclidean clustering and plotted in R as dotplots. A minimum of two genes with orthology per scaffold were considered. Ancestral bilaterian linkage groups were identified by clustering orthologs between *B. floridae* and *M. yessoensis*, identifying initially 19 linkage groups, largely corresponding to chordate linkage groups (CLGs [45]). BLAB and BLGB, both chordate fusions, are separated into BLGA1 and BLGA2 and BLGB1, BLGB2, and BLGB3, respectively (Supplementary Table 8). Using further comparisons to *N. vectensis* and other molluscs (Supplementary Table 8), we were able to identify that recent fusions observed in chromosome 2, 3, and 4 in *B. floridae* are also derived from distinct chromosomal units. Implementing these findings, we recovered 23 ancestral bilaterian linkage groups (BLGs, Supplementary Fig. 4, Supplementary Fig. 5).

In total 6,617 gene families were assignable to the 23 BLGs. This identity was used to profile BLG content along chromosomes (Supplementary Fig. 4), as previously described[45]. Briefly, BLG content was counted in windows of 10 orthologous genes, with the maximal spacing between the closest pair of genes of 5Mb.

Significance of BLG enrichment per chromosome was computed with one-sided Fisher test (Bonferroni multiple test corrected, alternative 'greater'). The number of shared orthologous gene families between two chromosomes was compared to the number of shared orthologs between all other chromosomes between two species. Number of shared orthologs and significance values were plotted with R (Supplementary Fig. 3, Supplementary Fig. 5). All code used for the synteny analysis can be found on https://bitbucket.org/viemet/public/src/master/CephChromosomes/.

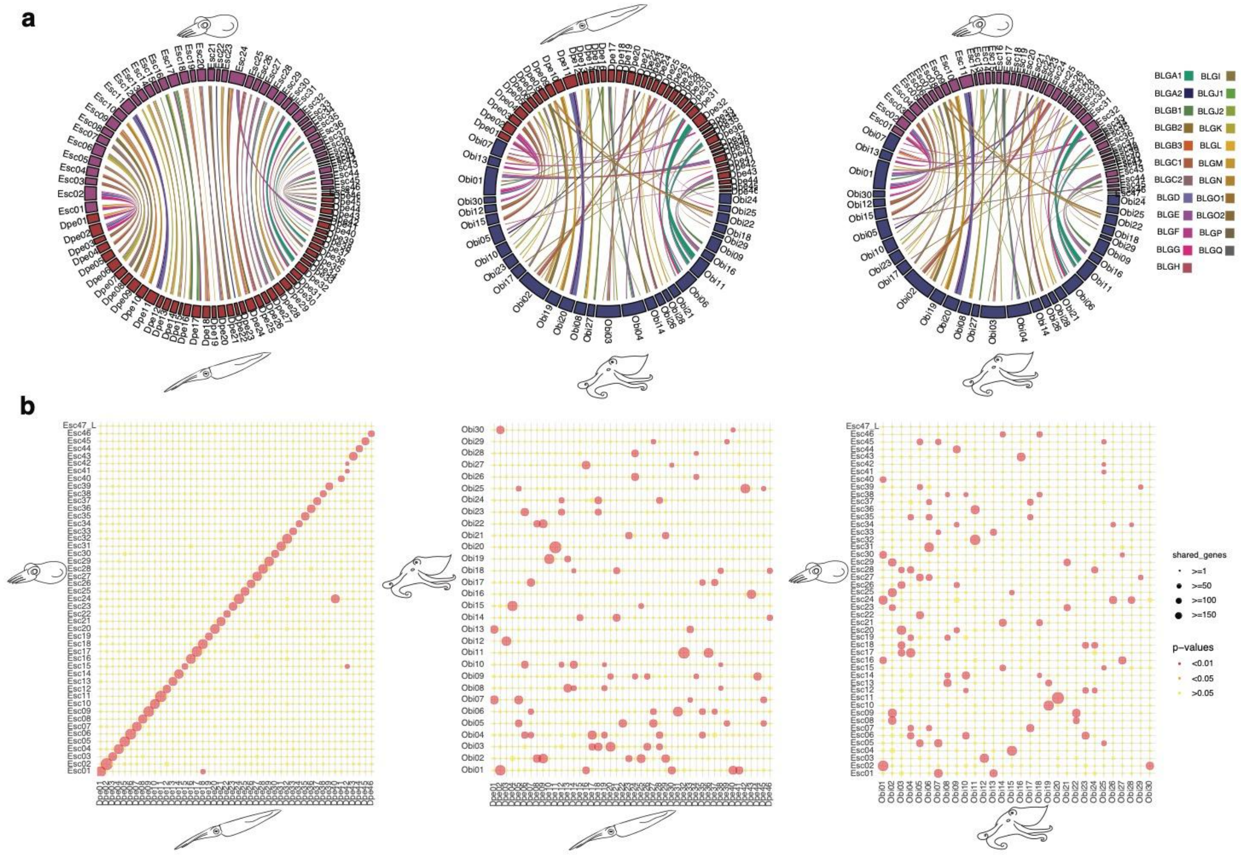

Supplementary Figure 3: Correspondence of cephalopod chromosomes between squid and octopus. a: Circos plots of shared orthologues between *D. pealeii*, *E. scolopes* and *O. bimaculoides* chromosomes. Chromosome sizes correspond to total number of shared orthologues, plotted are only orthologies with at least 10 shared genes per BLG.**b:** Fisher plots of pairwise chromosome correspondences.

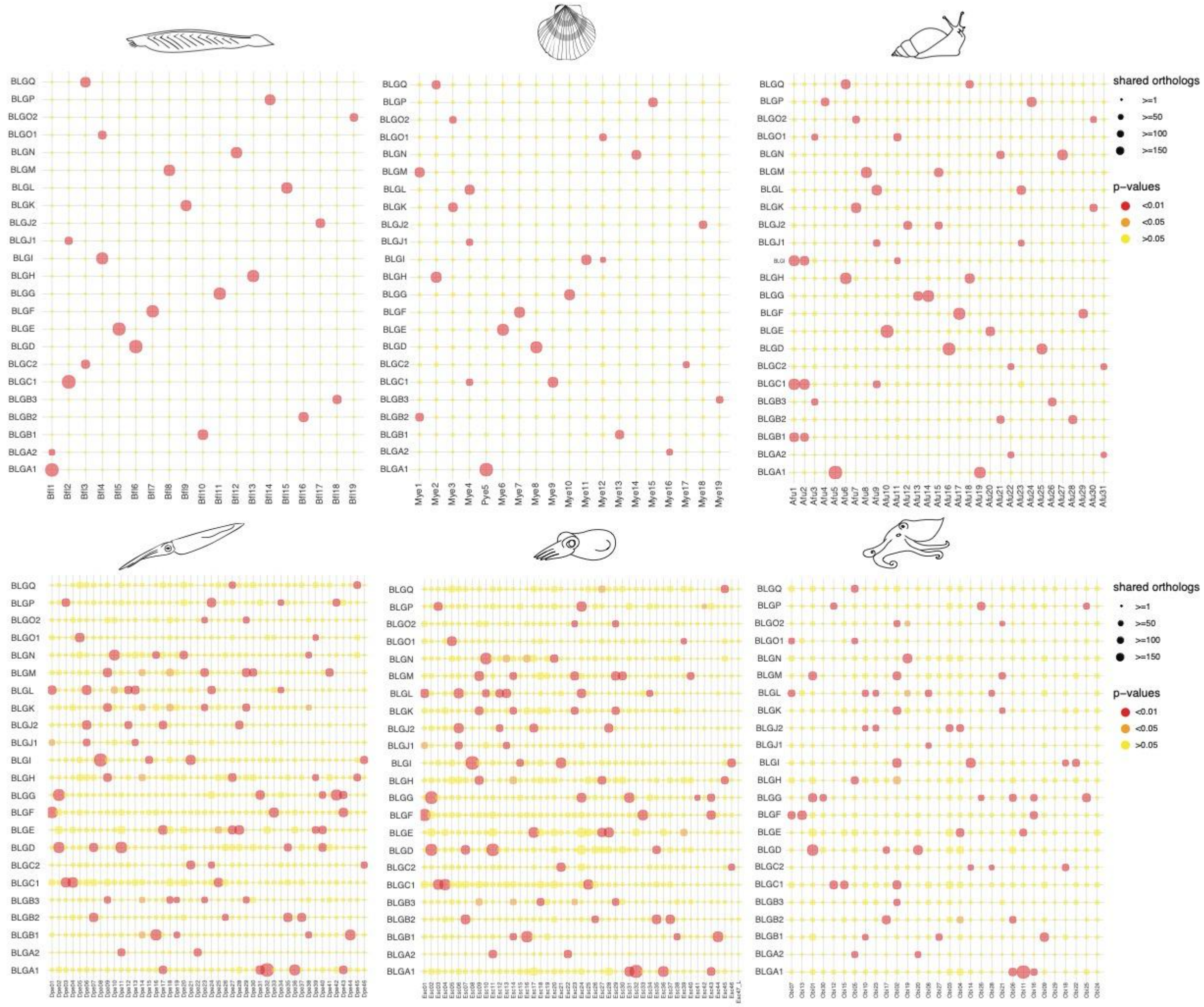

Supplementary Figure 4: **Fisher plots** (one-sided, alternative “greater”, Bonferroni multiple test corrected) showing enrichment of orthologs for each BLG on chromosomes of six species. Size of dots corresponds to the number of shared orthologs between a given BLG and the chromosome of a species. P-values are color coded. *B. floridae* chromosomes correspond 1:1 to the 23 BLGs, except for chromosomes 1-4, which underwent fusions of 2 BLGs. The majority of *M. yessoensis* chromosomes also correspond 1:1 to BLGs, except for chromosomes 1-4 and chromosome 12. *A. fulica* chromosomes show signs of fusions of up to three BLGs on 13 out of 31 chromosomes ($p<0.01$) and some chromosomes show signatures of whole genome duplication in this species. Squid (*D. pealeii* and *E. scolopes*) have few chromosomes that correspond to one BLG only and show fusions of up to 4 BLGs ($p <0.01$). The number of genes corresponding to different BLGs is widely distributed on each chromosome. Similar patterns are visible in *O. bimaculoides*, with one chromosome showing fusions of 6 BLGs (Obi02, $p<0.01$).

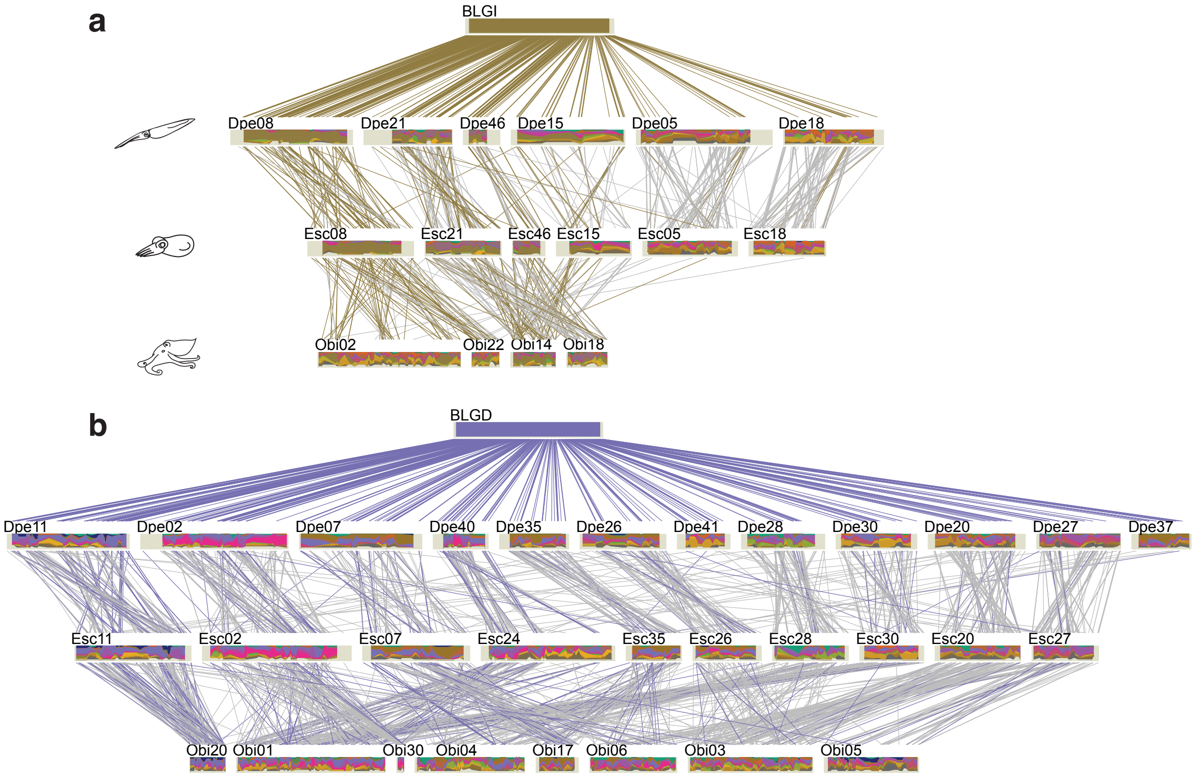

Supplementary Figure 5: Distribution of selected BLGs on cephalopod chromosomes. **a**: Distribution of BLGI on cephalopod chromosomes (min 10 BLGI orthologues). BLGI is distributed over several of chromosomes in each species. **b**: Distribution of BLGD on cephalopod chromosomes (min 10 BLGD orthologues). BLGD is highly fissioned and distributed on many chromosomes. Colored lines – orthologues of specific BLG, grey lines – other BLG orthologues between chromosomes.

Coleoid cephalopod linkage groups (CephLGs)

The number of CephLGs was inferred via counting of orthologous chromosomes in *D. pealeii* and *O. bimaculoides* with the least number of BLG combinations. Any homologous chromosome to this initial set that has additional BLG contribution is considered a secondary fusion. We calculated the list with a custom script and manual inspection of connectivity graphs of cephalopod chromosomes and their BLG contribution. In total we found 33 CephLGs, which we consider to be an underestimate as some combinations could not be clearly resolved. We provide the list of CephLG-like chromosomes and their BLG complement in Supplementary Table 9.

Colinearity in cephalopod and bird genomes

Mutual best hits (MBHs) were computed between *D. pealeii* and *E. scolopes* (13,365 MBHs) and zebrafinch (*Taeniopygia guttata*) and chicken (15,239 MBHs) (*Gallus gallus domesticus*)

(Fig. 3a, b) as described above. Dotplots were constructed with code described in the macrosynteny section. Alignments between selected chromosomal pairs were conducted with blastn megablast (-task megablast -perc_identity 0 -template_length 16 -penalty -2 -word_size 11 -template_type coding_and_optimal). For example, for Dpe12 and Esc12, 4,690 high-scoring alignment pairs were produced. Alignment coordinates were transformed into non-overlapping windows of 10kb (any alignment within a given window is sufficient to count that window) (Fig. 3c.) To quantify overall microsyntenic preservation, a published microsynteny pipeline[46] was run, allowing for up to 5 intervening genes (Nmax=3). The number of genes in microsyntenic runs was profiled. Median run length for *D. pealeii* - *E. scolopes* was 3 genes and zebrafinch-chicken 8 genes. Cumulatively, microsyntenic stretches of up to 11 genes or fewer in *D. pealeii* - *E. scolopes* make up to 90% of all genes, whereas runs of up to 68 genes make up the respective proportion in zebrafinch-chicken (Fig. 3d).

Supplementary Table 7: Datasets used in macrosynteny analysis

Species	Common name	Data access
Achatina fulica	Giant African snail	ftp://parrot.genomics.cn/gigadb/pub/10.5524/100001_101000/100647/
Anadara broughtonii	Blood clam	ftp://parrot.genomics.cn/gigadb/pub/10.5524/100001_101000/100607/
Mizuhopecten yessoensis	Yesso scallop	Wang et al, 2017
Nautilus pompilius	Emperor or pearly nautilus	Bioproject PRJNA614552
Octopus bimaculoides	California two-spot octopus	Albertin et al. 2015 + new assembly, this paper
Euprymna scolopes	Hawaiian bobtail squid	Belcaid et al. 2019, Schmidbaur et al., submitted
Doryteuthis pealeii	Longfin inshore squid	This paper
Branchiostoma floridae	Florida lancelet	Simakov et al, 2020
Nematostella vectensis	Starlet sea anemone	Putnam et al, 2007
Gallus gallus	Chicken	Ensembl, v5
Taeniopygia guttata	Zebra finch	Ensembl TaeGut1_v1

Supplementary Table 8: Reconstruction of linkage groups

BLG	*M. yessoensis*	*Achatina fulica*	*Anadara broughtonii*	*Nematostella vectensis*	"Ancestral"
BLGA	unfused	unfused	unfused	unfused	yes
BLGA2	unfused	C2	unfused	N	yes
BLGB	unfused	C, I	unfused	B2, B3	yes
BLGB2	N	unfused	unfused	B1, B3	yes
BLGB3	unfused	unfused	P	B1, B2	yes
BLGC	unfused	B, I	unfused	unfused	yes
BLGC2	unfused	A2	unfused	unfused/Q?	yes
BLGD	unfused	unfused	unfused	unfused	yes
BLGE	unfused	unfused	unfused	L	yes
BLGF	unfused	unfused	unfused	E?	yes
BLGG	unfused	unfused	unfused	J?/C?	yes
BLGH	Q	Q	Q	unfused	Molluscan fusion?
BLGI	unfused	B.C	unfused	O	yes
BLGJ	L	L	L	G	Molluscan fusion?
BLGJ2	unfused	unfused	unfused	Q	yes
BLGK	O2	O2	O2	C2	Molluscan fusion?
BLGL	J	J	J	E	Molluscan fusion?
BLGM	B2	unfused	unfused	unfused	yes
BLGN	unfused	unfused	unfused	N	yes
BLGO	unfused	unfused	unfused	I	yes
BLGO2	K	K	K	unfused	Molluscan fusion?
BLGP	unfused	unfused	B3	C2?	yes
BLGQ	H	H	H	J2	Molluscan fusion?

Supplementary Table 9. Composition of putative cephalopod linkage groups. Split BLGs are numbered.

CephLG No	BLG composition	Chromosomes
CephLG1	BLGA1	Obi11, Dpe36, Dpe32
CephLG2	BLGA1.2-BLGF1.1-BLGG1.1	Obi16, Dpe43
CephLG3	BLGA1.3-BLGG1.2	Dpe31
CephLG4	BLGA2.1-BLGD1.1	Obi20, Dpe11
CephLG5	BLGA2	Dpe22
CephLG6	BLGB1.1	Obi09, Dpe44, Dpe19
CephLG7	BLGB1.2	Obi27, Dpe16
CephLG8	BLGB2.1	Dpe26, Dpe37
CephLG9	BLGB2.2-BLGD1.2	Obi17, Dpe07, Dpe35
CephLG10	BLGB3.1-BLGH1.1-BLGM1.2-BLGK1.1	Dpe09
CephLG11	BLGC1.1-BLGP1.1	Obi12, Dpe03
CephLG12	BLGC1.2	Obi15, Dpe04, Dpe25
CephLG13	BLGC2.1-BLGI1.3	Obi18, Obi14, Dpe21, Dpe46
CephLG14	BLGD1.3-BLGG1.4	Dpe02, Dpe40
CephLG15	BLGE1.1-BLGJ2.2	Dpe28
CephLG16	BLGF1.2	Obi13, Dpe33
CephLG17	BLGG1.3	Obi30
CephLG18	BLGH1.2-BLGQ1.1	Dpe45
CephLG19	BLGI1.1	Obi22, Dpe08
CephLG20	BLGI1.2	Dpe15
CephLG21	BLGJ1.1-BLGL1.2	Obi08, Dpe13
CephLG22	BLGJ2.1	Obi03
CephLG23	BLGK1.2-BLGM1.2-BLGO2.1	Obi21
CephLG24	BLGL1.1-BLGP1.2	Dpe34
CephLG25	BLGM1.3	Dpe41, Dpe30
CephLG26	BLGN	Dpe20
CephLG27	BLGO1.1	Dpe05
CephLG28	BLGB1.3-BLGH1.3	Dpe14
CephLG29	BLGB3.2-BLGH1.4-BLGK1.3-BLGM1.4	Dpe29, Dpe23
CephLG30	BLGC2.2-BLGL1.3	Obi28
CephLG31	BLGG1.5-BLGP1.3	Obi26, Obi25, Dpe42
CephLG32	BLGJ2.3-BLGL1.4	Obi23, Dpe12

Supplementary Table 10. List of squid chromosomes that correspond 1:1 or 1:2 to octopus chromosomes.

Squid chromosome	Corresponding octopus chromosome
Esc11/Dpe11	Obi20
Esc03/Dpe03	Obi12
Esc43/Dpe43	Obi16
Esc32&36/Dpe32&36	Obo11

Supplementary Note 6: Large gene families

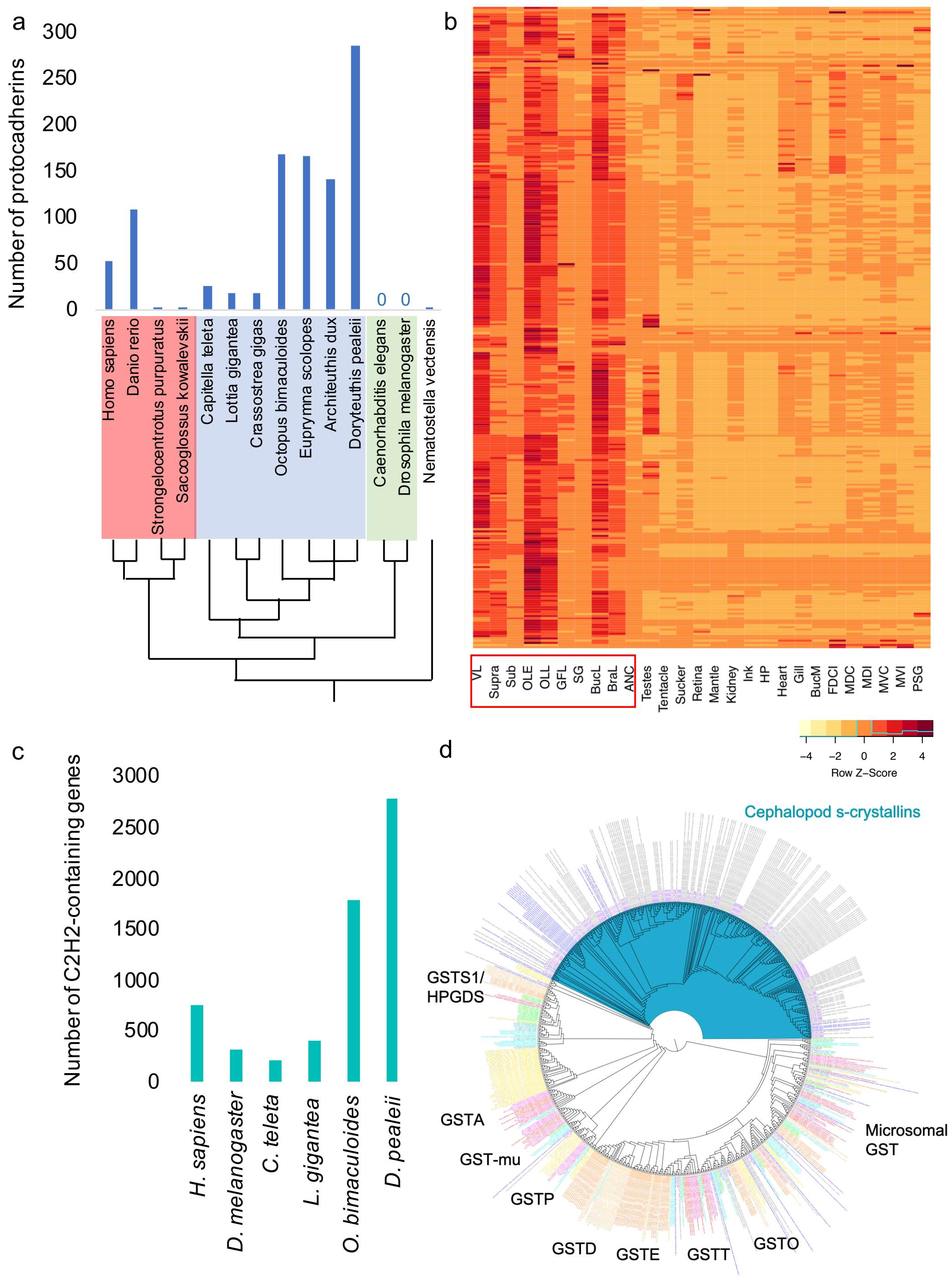

Supplementary Figure 6: Large gene families in cephalopod genomes. a: Number of protocadherin genes in selected metazoan genomes. Phylogenetic tree below indicates the relationship of the species represented. b: Expression profiles of 288 protocadherins across *D. pealeii* transcriptomes demonstrates elevated expression in some neural tissues. Cells are colored according to standard deviation from the mean expression level, neural tissues are

indicated in the red box. Abbreviations: VL: vertical lobe, Supra: supraesophageal brain, Sub: subesophageal brain, OLE: optic lobe early, OLL: optic lobe late, GFL: giant fiber lobe, SG: stellate ganglion, BucL: buccal lobe, BraL: brachial lobe, ANC: axial nerve cord; HP: hepatopancreas; BucM: buccal mass, FDCI: dorsal fin skin, MDC: dorsal mantle chromatophore skin layer, MDI: dorsal mantle iridophore skin layer, MVC: ventral mantle chromatophore skin layer, MVI: ventral mantle iridophore skin layer, PSG: posterior salivary gland. c: C2H2 zinc-finger gene family content in metazoans highlights an expansion in cephalopod genomes. d: Phylogeny of glutathione S-transferase genes in metazoans. GSTs identified in human (red), mouse (pink), *Drosophila melanogaster* (orange), *Tribolium castaneum* (peach), *Caenorhabditis elegans* (yellow), *Capitella teleta* (green), *Lottia gigantea* (teal), *Crassostrea gigas* (sky blue), *Octopus bimaculoides* (dark blue), *Euprymna scolopes* (purple), and *Doryteuthis pealeii* (gray). The cephalopod s-crystallin expansion are highlighted in turquoise.

Supplementary Note 7: RNA-editing

Identification and classification of confident transcriptome variants

RNA-edited sites were identified from the RNA-seq genome alignments of a total of 25 samples, from 24 different tissues, dissected from the same *D. pealeii* genome-reference individual (specimen A, Supplementary Table 3). The Optic Lobe had a technical replicate, for which one sample was collected early and late during the dissection process (OLe and OLl, respectively). All samples were mapped against the squid reference genome (online methods). All samples were analyzed for RNA editing with the exception of the ink sample due to the high percentage of duplicated reads (Supplementary Table 3). During the variant calling process we applied stringent base and mapping quality filters to avoid false-positive RNA-variant calls caused by RNA-seq read misalignments, sequencing errors, or by the presence of genomic variants. In short, we required transcript variants to have a minimum of 10 reads depth per site, mapping quality of alignments above 30, and a minimum of 0.1% in at least one of the 25 samples. Transcriptome variants overlapping with genomic variants were excluded (see online methods). Given the number of Adenosines present in the different genomic features (Supplementary Table 14), we find that 3'UTR and coding sequences (recoding and synonymous) are preferentially targeted by ADAR proteins, and that Adenosines in 3'UTR are in repetitive regions (Supplementary Fig. 8b).

Using our 25 samples (excluding ink) we found 77,082 recoding edits in 8,891 genes (median of 4 and mean of 8.7 edits per gene) (Supplementary Table 12). In order to compare the degree of biological and technical reproducibility we retrieved 11 transcriptomes (5 neural: BGr, GFLr, OLr, SGr, and VLr; and 6 non-neural: BHr, Car, Gilr, ILr, MELPr, and VELPr) from the study of Alon et al. using a different *D. pealeaii* specimen[47] and analyzed it with our mRNA editing identification pipeline (Supplementary Table 12). Using Alon et al.'s data with our pipeline, we found 50,320 recoding sites in 7,336 genes (using Alon, et al[47]). These numbers are close to those reported by the genome-free RNA-editing analysis previously presented by Liscovitch-Brauer (2017) where they found 54,287 recoding edits in 6,688 ORFs; differences presumably arise from differences between the genome-free and genome-anchored methods for identifying edited sites. Comparing the analysis of the two datasets with our pipeline, we find that the number of CDS edited sites (recoding plus synonymous) per gene are highly correlated (Supplementary Fig. 7a). Likewise, the edit frequencies between homologous samples of separate specimens also strongly correlate (R>0.84). This correlation is higher for technical replicates (R>0.94, between OLe and OLl) (Supplementary Fig. 7b).

Given the diversity of collected tissues, we aimed to classify edit sites in two ways: one defines edit preference across tissues (edit types) and another one that determines the robustness of mRNA editing (Supplementary Table 13). To define edit types we used the projections of weighted editing averages for neural (WN) and non-neural samples (WNN) to classify ADAR targets into four edit types (see online methods): 1) Ubiquitous-Low: edit frequency of WN and WNN is below 5%, 2) Ubiquitous-High: WN above 60% and WNN are above 40%, 3) Ubiquitous-Medium: WNN between 5-40%, WN between 5-60%, and the ratio between WN and

WNN was below 2.75 , 4) Neural: WN/WNN ratio is above 2.75, WNN below 40% and WN below 60% (Fig. 5d, Supplementary Fig. 8)). Edit type was assigned to sites with cumulative read depth of more than 10 reads for neural and non-neural samples, otherwise the sites were unclassified. The second classification of sites used were by robustness, for which “robust” sites are those where at least one of the 25 samples has more than 25% edit frequency.

The expression of any of the four *ADAR* genes does not appear to explain the differences in editing profiles across tissue types (Fig. 7). Unlike *ADAR2* that is expressed and edited in all tissues, *ADAR1* is predominantly expressed in neural tissues and other non-neural tissues (MDC and Testis), yet editing only is detected in neurally-derived tissues, including the retina and ANC. Suggesting that ADAR1 by itself is not sufficient to explain the presence of neural-type edits and may require other neural-specific RNA binding proteins (*e.g. ELAVL1/4* and *CELF* homologs) to efficiently edit its targets (Fig. 7 and Supplementary Figs. 10 and 11) To observe how edit frequencies vary across tissues and how edit types are distributed across the mRNA, we examined the editing profile of constitutively expressed genes with diverse biological functions (Supplementary Fig. 12) We observe that transcripts can have different combinations of edit types throughout the gene body. In some cases, such as *GRIK, KCNA1, CHRNA4*, and *AP3M1*, the ubiquitous-high edits (edited high in both neural and non-neural samples) are in close proximity to other robust edits (ubiquitous-high, ubiquitous-medium and neural types), while ubiquitously-low (non-robust) edits are dispersed through the transcript (Supplementary Figs. 11 and 12). This could suggest that ubiquitously-high edits are in hyper-stable dsRNAs regions that lead to efficient binding and deamination by ADAR proteins. In contrast, edits that are edited mainly in neural tissues (e.g. neural-type edits in *MYCBP2, ARGAP32*) may be in relatively less stable dsRNA regions and may require the interaction of RNA editing cofactors. The wide distribution and predominance of ubiquitous-low edit types could come from stochastic editing in not-so-favorable dsRNA structures that are promiscuously targeted by ADAR.

To ascertain the degree of evolutionary conservation among edited *vs* non-edited adenosines in coding regions, we aligned genomic shotgun sequence reads from related squid against the reference *D. pealeii* genome. We used the genomic shotgun sequence from another *D. pealeii* specimen (from the same Woods Hole population) to focus on sites that are likely to be fixed as adenosine in *D. pealeii*[47]. We also generated new genomic reads for the California market squid *D. opalescens* and a more distantly related loliginid *H. bleekeri* from Japan (Supplementary Note 2). The median synonymous divergences of these two genomes relative to *D. pealeii* are 3.75% and 13.55%, respectively. For each organism, we selected high-quality variant calls overlapping CDS in the *D. pealeii* genome. We required a minimum mapping quality above 30 and sequence depth within the expected depth within CDS regions. The expected coverage range was determined from the mean ±2 standard deviations of shotgun coverage at CDS regions (Supplementary Fig. 1). In order to determine the ancestral genotype of edited and unedited adenosines, we focused on genomically homozygous adenosines in *D. pealeii* (both specimens) and counted the genotype calls from *D. opalescens* and *H. bleekeri* (Supplementary Tables 15 and 16). For genotypes where all three species conserve the homozygous adenosines, the recoding:synonymous ratio is slightly higher for robust edits compared to sites that are not edited. We also considered sites where *D. pealeii* is A/A in both specimens but *D.*

opalescens and *H. bleekeri* are homozygous for other nucleotides (*e.g.* G/G, T/T or C/C). These are presumably sites that became fixed for the A allele in the *D. pealeii* lineage after it diverged from *D. opalescens*, with *H. bleekeri* serving as the outgroup to polarize the change. For such sites the ratio of recoding:synonymous is not significantly different for non-edited and robustly edited adenosines in *D. pealeii* (Supplementary Table 15). The same analysis focused exclusively on robustly edited sites in *D. pealeii* shows that only the conserved adenosines in the Ubiquitous High category has a significantly higher rate of recoding edits over synonymous changes compared to the other edit types (Supplementary Table 17). Although the number of edits under this category is low (n=132) these data suggest that recoding edits that are heavily edited are evolving under positive selection.

A database of all annotated ADAR target sites in the squid genome can be found at https://github.com/sofiamedinaruiz/Squid_RNAediting and at https://doi.org/10.6078/D15X38.

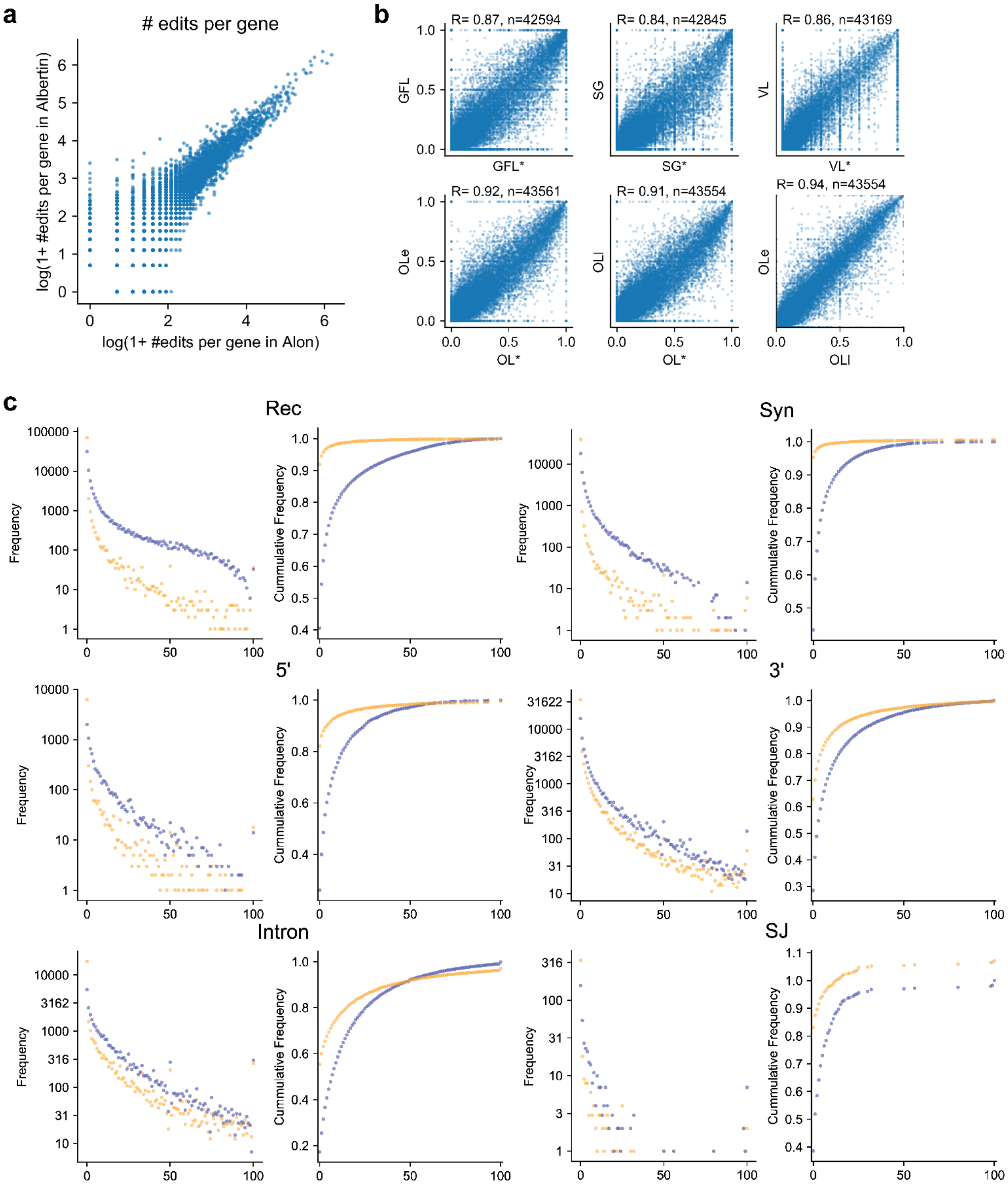

Supplementary Figure 7: Editing frequency across tissues and gene elements. a: Number of recoding and synonymous edits per gene from datasets from independent squid specimen (Alon et al 2015, x-axis) compared to the collection obtained in this study (y-axis). b: Edit frequencies obtained from biological and technical replicates are highly correlated. Scatter plots compare the edit frequencies of tissues from two independent sample collections (asterisk denotes samples obtained by Alon et al., 2015). The bottom right plot compares two technical replicates obtained from the same individual. The number of common sites between the pair samples is displayed at the top of the plots (n). c: The majority of edits have an edit frequency below 1% (e.g. >40% and >90% of weighted neural samples and non-neural samples, respectively). Histogram of edit frequencies contrasting the weighted edit frequency between Neural and Non-neural samples for edits overlapping different gene portions. Recoding and synonymous edits show the greatest distribution differences.

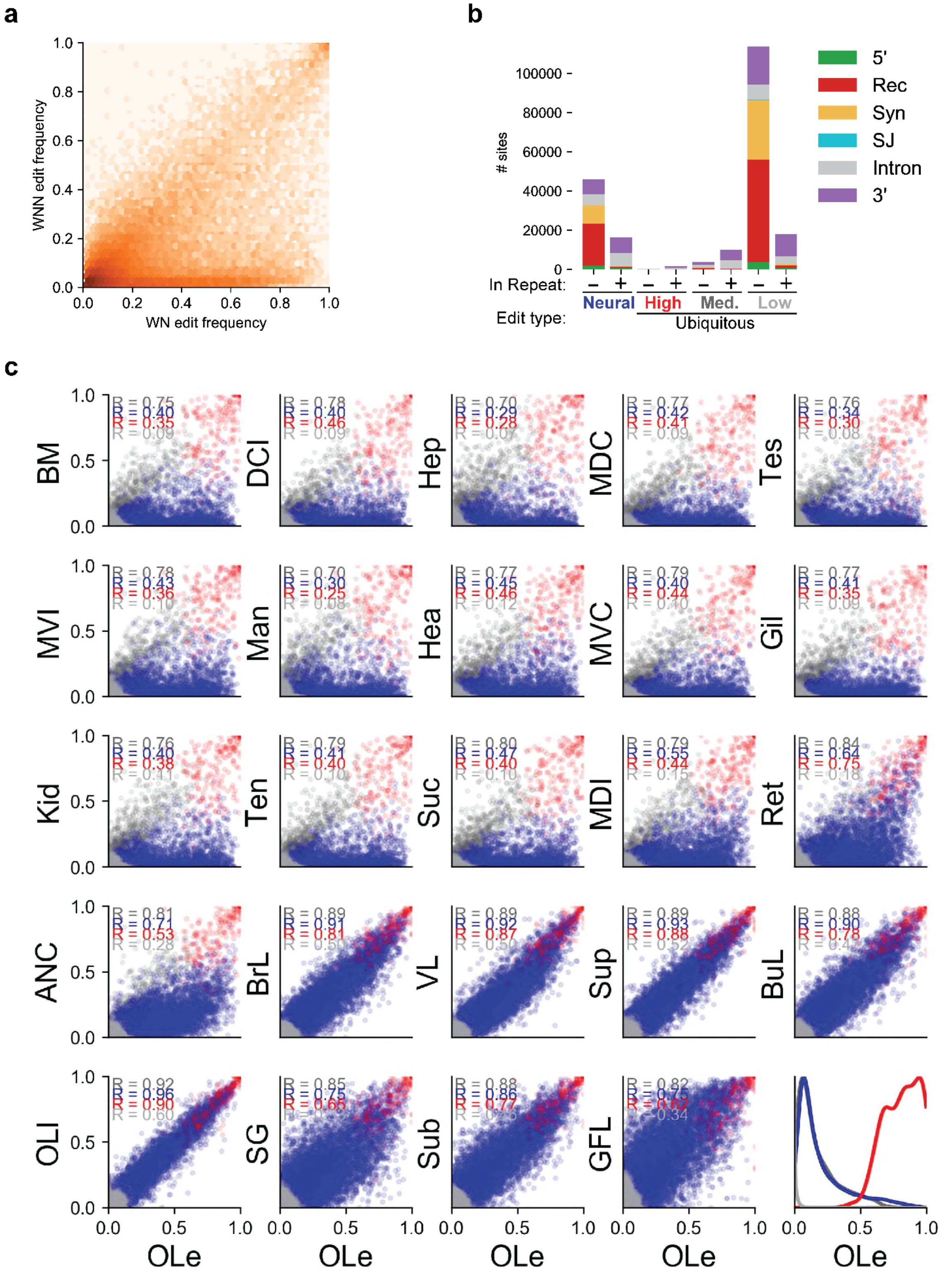

Supplementary Figure 8: Comparison of editing across tissue and editing types. a: Density histogram for weighted neural (WN) and weighted non-neural (WNN) (n=35,857 sites

with aggregated read depth greater than 10 reads and edit frequencies above 1%). b: Coding edits are found predominantly in Neural and Ubiquitously low edit types. 197,549 sites with at least 10 reads of depth in neural samples and non-neural samples. Weighted edit frequencies for neural (WN) and non-neural (WNN) samples classified by genic locations. c: Scatter plots of edit frequencies comparing one of the two samples obtained from optic lobe (OLe) against all other tissues.

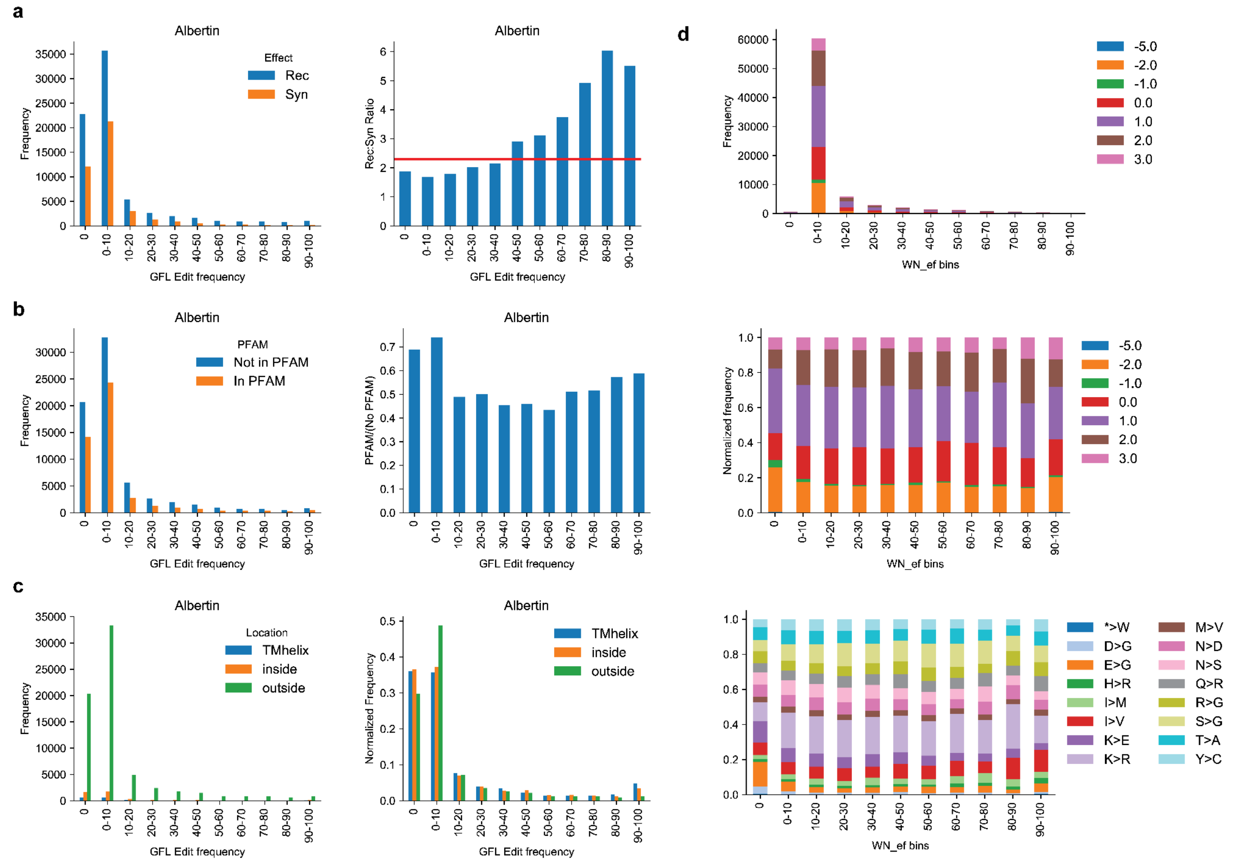

Supplementary Figure 9: Editing in PFAM domains, transmembrane domains, and across blosum scores. Variation in diverse quantities stratified by the Giant Fiber Lobe edit frequency (GFL, panels a-c) or weighted neural edit frequency (WN_ef, panel d), in bins of 10% edit frequency. a: Counts of recoding vs synonymous edits per edit frequency bins. The red line on the right panel represents the ratio of recoding over synonymous edits of non-edited Adenosines in the genome to represent the expected ratio. b: Counts of sites overlapping PFAM domains. c: Counts of sites overlapping transmembrane domains (left) and proportion of transmembrane annotations that are edited with respect to the total length of each. d: Counts of the blosum62 scores of recoding edits: absolute counts (top), normalized (middle). The normalized counts of amino acid type substitution on the bottom.

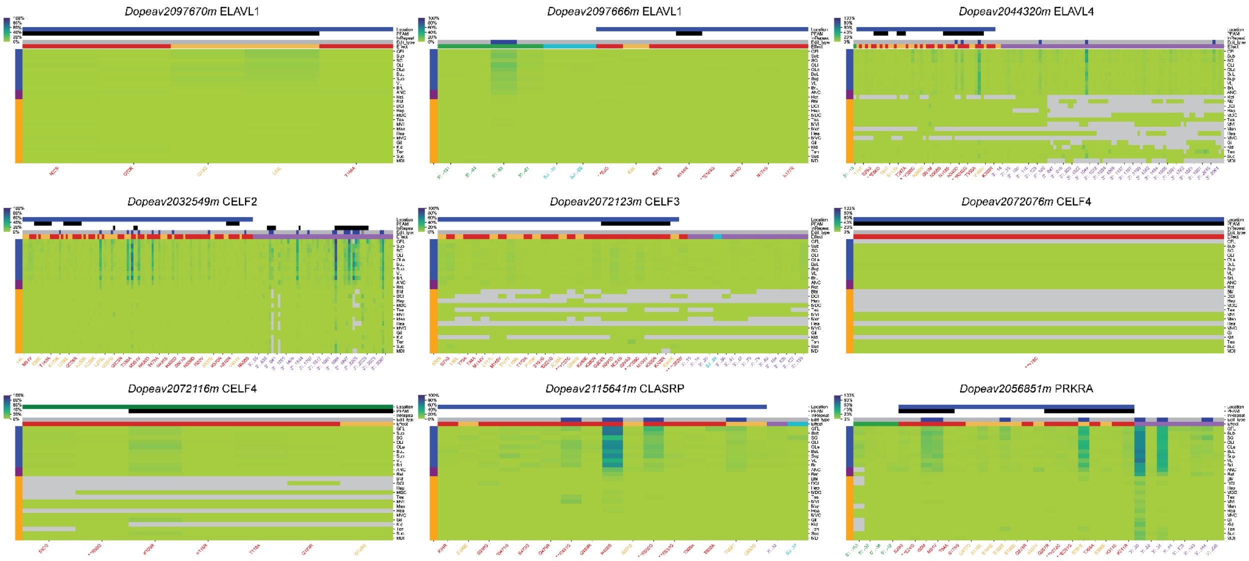

Supplementary Figure 10: mRNA editing profile of mRNA-associated proteins.

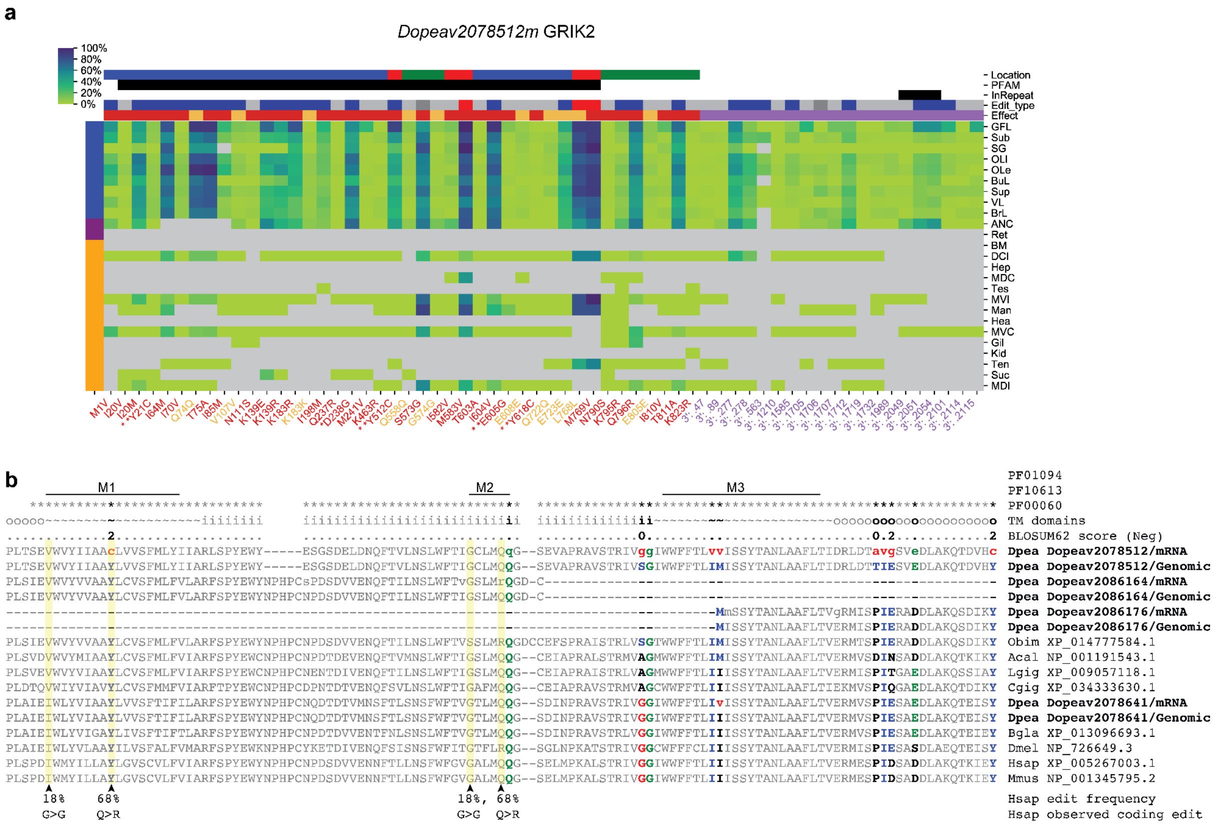

Supplementary Figure 11: Editing in GRIK. a: Editing profile of GRIK (Dopeav2078512m) gene. b: Multiple sequence alignment of GRIK homologs. Species considered for the alignment: 6 molluscs: Squid (reference *D. pealeii*, Dpea), octopus (*Octopus bimaculoides*, Obim), sea hare *Aplysia californica* (Acal), owl limpet *Lottia gigantea* (Lgig), Pacific oyster (Cgig), snail

Biomphalaria glabrata (Bgla); one insect: fruit fly *Drosophila melanogaster* (Dmel); and two mammals: mouse (Mmus) and human (Hsap). All peptide sequences derived from the genome sequence with the exception of squid peptide(s), which derive from messenger RNA (mRNA). Amino acids overlapping edit sites in squid mRNA appear in lower case. The alignment highlights amino acids as follows: Genomic-encoded squid amino acid (blue), recoded (red), and synonymous (green). Non-squid amino acids aligned to edits in squid inherit the corresponding amino acid color or else appear in black. The top of the alignment is highlighting the three first transmembrane domains from human GRIK2 (M1, M2, M3). Shared edit sites between mouse and human are highlighted in yellow. The percentage of editing and the amino acid change reported in human is displayed below the alignments.

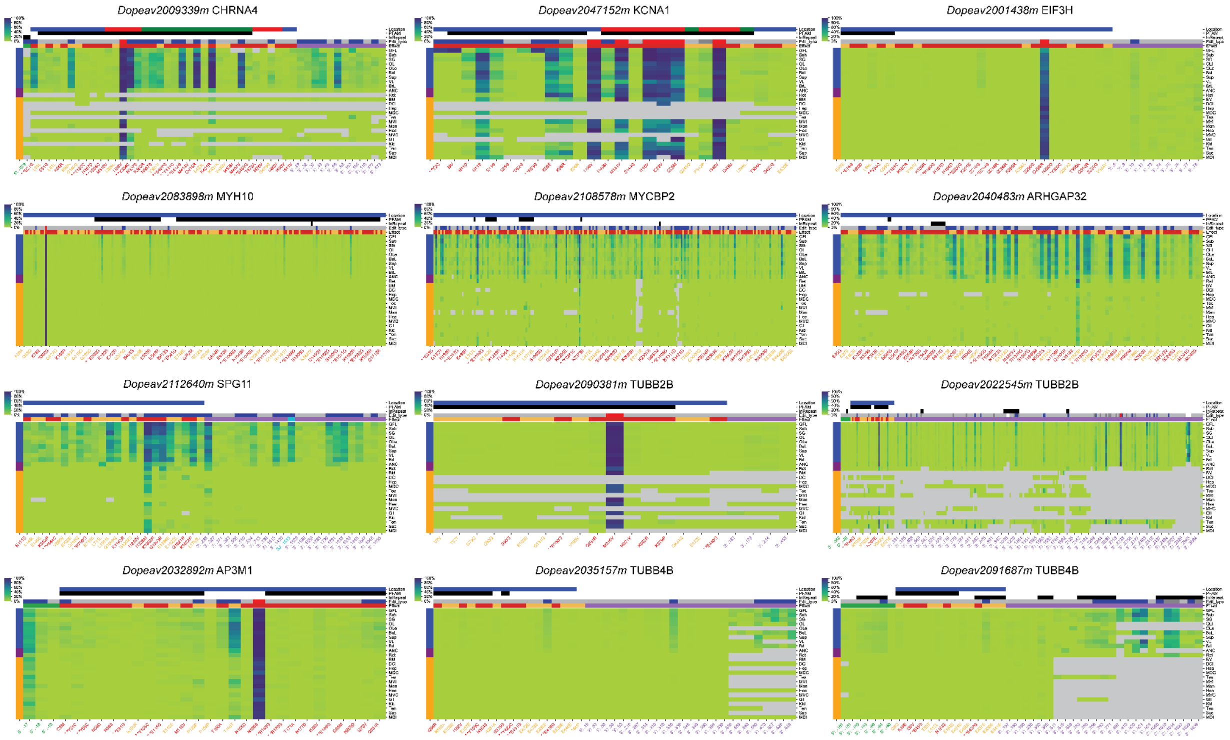

Supplementary Figure 12: Editing profiles from genes with diverse biological functions. Transmembrane receptors: CHRNA4 (Dopeav2009339m) and KCNA1 (Dopeav2047152m). Transcription factors: EIF3H (Dopeav2001438m). Ubiquitin-ligase: MYCBP2 (Dopeav2108578m), Myosin: MYH10 (Dopeav2083898m). Rho GTPase: ARHGAP32 (Dopeav2040483m). Vesicle trafficking: SPG11 (Dopeav2112640m), AP3M1 (Dopeav2032892m). Tubulin genes: TUBB4B (Dopeav2091687m, Dopeav2035157m) and TUBB2B (Dopeav2022545m).

Supplementary Table 11. Number of edit sites identified the genome.

	Robustness	
Effect	Robust	Not robust
5'	2,207	5,555
Rec	15,293	61,789
Syn	5,528	35,806
SJ	119	325
Intron	16,738	15,914
3'	16,635	38,108
Intergenic	149,098	227,050
Total	205,618	384,547

Supplementary Table 12: Comparison of editing in previous and current study. Number of RNA edit sites identified from two *D. pealeaii* transcriptome collections using the analysis pipeline described in online methods. 11,841 genes in the current study contain any kind of edit. 9,960 genes with at least one edit (any type, coding) 5,905 genes have at least one robust edit 4,055 genes have no robust edits.

	Alon et al., 2015		Current study	
Effect	# edits	# genes	# edits	# genes
3'	34,991	3,718	54,743	4,493
5'	6,137	1,942	7,762	2,053
Intron	16,477	2,017	32,652	2,698
Rec	50,320	7,336	77,082	8,891
SJ	285	220	444	334
Syn	29,480	6,820	41,334	7,482
Total	137,690	10,659	214,017	11,841

Supplementary Table 13. The number of edit sites by robustness and edit type. Unclassified edits belong to sites with insufficient cumulative read depth (<10 reads) in Neural samples or Non-neural samples.

		Effect						
Robustness	Edit_type	5'	Rec	Syn	SJ	Intron	3'	Sum
Robust	Neural	1,646	13,965	4,854	78	8,728	9,467	38,738
	Ubiq-High	56	206	28	10	1,049	1,022	2,371
	Ubiq-Low	101	414	231	6	537	609	1,898
	Ubiq-Med	322	519	355	22	4,924	5,327	11,469
	Unclassified	82	189	60	3	1,500	210	2,044
	Sum robust	2,207	15,293	5,528	119	16,738	16,635	56,520
Not robust	Neural	1,024	8,303	4,641	42	3,568	6,175	23,753
	Ubiq-Low	4,368	53,182	30,971	268	11,099	30,273	130,161
	Ubiq-Med	60	139	123	11	642	1,518	2,493
	Unclassified	103	165	71	4	605	142	1,090
	Sum not robust	5,555	61,789	35,806	325	15,914	38,108	157,497
TOTAL		7,762	77,082	41,334	444	32,652	54,743	214,017

Supplementary Table 14: **Quantification of expressed Adenosines segregated by genomic features.**

	**Edited As**	**Not edited As**	**Sum As**	**% edited**
**5'**	7,762	602,413	610,175	1.27%
**Rec**	77,082	2,042,141	2,119,223	3.64%
**Syn**	41,334	869,070	910,404	4.54%
**SJ**	444	43,249	43,693	1.02%
**Intron**	32,652	6,192,113	6,224,765	0.52%
**3'**	54,743	965,035	1,019,778	5.37%
**Total**	214,017	10,714,021	10,928,038	1.96%

Supplementary Table 15. Number of edited sites by genomic location, edit robustness and repeat overlap.

		Repeat overlap		% overlap w/repeats
**Effect**	**Robustness**	**Yes**	**No**	
**3'**	Not robust	14,940	23,168	39.20%
	Robust	10,874	5,761	65.37%
**5'**	Not robust	1,033	4,522	18.60%
	Robust	815	1,392	36.93%
**Intron**	Not robust	6,075	9,839	38.17%
	Robust	10,978	5,760	65.59%
**Rec**	Not robust	1,640	60,149	2.65%
	Robust	547	14,746	3.58%
**SJ**	Not robust	51	274	15.69%
	Robust	30	89	25.21%
**Syn**	Not robust	1,001	34,805	2.80%
	Robust	189	5,339	3.42%
**Intergenic**	Not robust	98,207	128,843	43.25%
	Robust	100,639	48,459	67.50%

Supplementary Table 16: Identification of new and conserved robust editing sites in *D. pealeii*. Homozygous genotypes from *Doryteuthis opalescens (Dopal)* and *Heterololigo bleekeri (Hbl)* from sites overlapping homozygous Adenosines in *D. pealeii*. Sites are subclassified by whether the Adenosine site in *D. pealeii* is not-edited, robustly edited (at least one sample presenting >25% editing), and not-robustly edited (edit below 25% in all samples). The frequency of sites that lead to recoding or synonymous changes is reported and used to determine the dN/dS ratio. The p-values obtained from the chi-square on each genotype combination.

Genotype (*Dopal, Hbl*)	A's in *Dpeal*	Effect			p-val (Chi-sqr per Genotype)
	Robustness	Rec	Syn	Rec:Syn	
A/A,A/A 99.25%	Not edited	3487181	1292934	2.697	7.10E-08
	Not robust	48206	25824	1.867	0.00E+00
	Robust	11643	3683	3.161	3.20E-18
C/C,C/C 0.14%	Not edited	3912	2974	1.315	9.60E-01
	Not robust	46	29	1.586	4.30E-01
	Robust	4	5	0.8	4.50E-01
G/G,G/G 0.47%	Not edited	8468	14437	0.587	6.30E-01
	Not robust	62	209	0.297	2.00E-06
	Robust	28	40	0.7	4.60E-01
T/T,T/T 0.14%	Not edited	3004	3581	0.839	9.50E-01
	Not robust	30	42	0.714	5.00E-01
	Robust	4	4	1	8.00E-01

Supplementary Table 17: New and conserved types of editing sites in *D. pealeii.* Similar to Supplementary Table 16 now subclassified by Edit type instead of robustness.

Genotype (*Dopal, Hbl*)	A's in *Dpeal*	Effect			p-val (Chi-sqr per Genotype)
	Edit_type	Rec	Syn	Rec:Syn	
A/A,A/A	Neural	17316	6743	2.568	2.44E-03
	Not-edited	3487181	1292934	2.697	6.89E-08
	Ubiq-High	124	8	15.5	5.04E-08
	Ubiq-Low	41924	22572	1.857	0.00E+00
	Ubiq-Med	290	119	2.437	3.78E-01
C/C,C/C	Neural	8	9	0.889	4.16E-01
	Not-edited	3912	2974	1.315	9.65E-01
	Ubiq-Low	41	25	1.64	3.86E-01
G/G,G/G	Neural	28	56	0.5	5.08E-01
	Not-edited	8468	14437	0.587	6.27E-01
	Ubiq-High	2	0	inf	6.39E-02
	Ubiq-Low	55	185	0.297	8.03E-06
	Ubiq-Med	4	8	0.5	8.03E-01
T/T,T/T	Neural	6	7	0.857	9.67E-01
	Not-edited	3004	3581	0.839	9.51E-01
	Ubiq-Low	27	39	0.692	4.46E-01
	Ubiq-Med	1	0	inf	2.75E-01

Supplementary Table 18: New and conserved types of robust edit sites in *D. pealeii.*
Similar to Supplementary Table 17 considering robust edit sites.

Genotype (*Dopal, Hbl*)	*Robust edits	Effect			p-val (Chi-sqr per Genotype)
	Edit_type	Rec	Syn	Rec:Syn	
A/A,A/A	Neural	10883	3420	3.182	7.05E-01
	Ubiq-High	124	8	15.5	1.00E-06
	Ubiq-Low	289	135	2.141	1.73E-04
	Ubiq-Med	233	87	2.678	1.89E-01
C/C,C/C	Neural	4	4	1	7.52E-01
	Ubiq-Low	0	1	0	3.71E-01
G/G,G/G	Neural	22	28	0.786	5.94E-01
	Ubiq-High	2	0	inf	8.52E-02
	Ubiq-Low	1	5	0.2	2.38E-01
	Ubiq-Med	2	7	0.286	2.69E-01
T/T,T/T	Neural	3	3	1	1.00E+00
	Ubiq-Low	0	1	0	3.17E-01
	Ubiq-Med	1	0	inf	3.17E-01

Supplementary References

1. Fiorito, G. *et al.* Guidelines for the Care and Welfare of Cephalopods in Research -A consensus based on an initiative by CephRes, FELASA and the Boyd Group. *Lab. Anim.* **49**, 1–90 (2015).
2. Fiorito, G. *et al.* Cephalopods in neuroscience: regulations, research and the 3Rs. *Invert. Neurosci.* **14**, 13–36 (2014).
3. Lopes, V. M. *et al.* Cephalopod biology and care, a COST FA1301 (CephsInAction) training school: anaesthesia and scientific procedures. *Invertebr. Neurosci. IN* **17**, 8 (2017).
4. Georganas, E. *et al.* Extreme-Scale De Novo Genome Assembly. *ArXiv170511147 Cs* (2017).
5. Ye, C., Hill, C. M., Wu, S., Ruan, J. & Ma, Z. (Sam). DBG2OLC: Efficient Assembly of Large Genomes Using Long Erroneous Reads of the Third Generation Sequencing Technologies. *Sci. Rep.* **6**, 31900 (2016).
6. Chin, C.-S. *et al.* Nonhybrid, finished microbial genome assemblies from long-read SMRT sequencing data. *Nat. Methods* **10**, 563–569 (2013).
7. Chaisson, M. J. & Tesler, G. Mapping single molecule sequencing reads using basic local alignment with successive refinement (BLASR): application and theory. *BMC Bioinformatics* **13**, 238 (2012).
8. Kajitani, R. *et al.* Efficient de novo assembly of highly heterozygous genomes from whole-genome shotgun short reads. *Genome Res.* **24**, 1384–1395 (2014).
9. Putnam, N. H. *et al.* Chromosome-scale shotgun assembly using an in vitro method for long-range linkage. *Genome Res.* (2016) doi:10.1101/gr.193474.115.
10. Durand, N. C. *et al.* Juicebox Provides a Visualization System for Hi-C Contact Maps with Unlimited Zoom. *Cell Syst.* **3**, 99–101 (2016).

11. English, A. C. *et al.* Mind the Gap: Upgrading Genomes with Pacific Biosciences RS Long-Read Sequencing Technology. *PLOS ONE* **7**, e47768 (2012).

12. Li, H. Minimap2: pairwise alignment for nucleotide sequences. *Bioinformatics* **34**, 3094–3100 (2018).

13. Li, H. Aligning sequence reads, clone sequences and assembly contigs with BWA-MEM. *ArXiv13033997 Q-Bio* (2013).

14. Garrison, E. & Marth, G. Haplotype-based variant detection from short-read sequencing. *ArXiv12073907 Q-Bio* (2012).

15. Gao, Y. M. & Natsukari, Y. Karyological Studies on Seven Cephalopods. *Venus Jpn. J. Malacol.* **49**, 126–145 (1990).

16. Wang, J. & Zheng, X. Comparison of the genetic relationship between nine Cephalopod species based on cluster analysis of karyotype evolutionary distance. *Comp. Cytogenet.* **11**, 477–494 (2017).

17. Albertin, C. B. *et al.* The octopus genome and the evolution of cephalopod neural and morphological novelties. *Nature* **524**, 220–224 (2015).

18. Schmidbaur, H. *et al.* Emergence of novel cephalopod gene regulation and expression through large-scale genome reorganization. (in revision).

19. Burton, J. N. *et al.* Chromosome-scale scaffolding of de novo genome assemblies based on chromatin interactions. *Nat. Biotechnol.* **31**, 1119–1125 (2013).

20. Belcaid, M. *et al.* Symbiotic organs shaped by distinct modes of genome evolution in cephalopods. *Proc. Natl. Acad. Sci.* **116**, 3030 (2019).

21. Servant, N. *et al.* HiC-Pro: an optimized and flexible pipeline for Hi-C data processing. *Genome Biol.* **16**, 259 (2015).

22. Zhang, G. *et al.* The oyster genome reveals stress adaptation and complexity of shell formation. *Nature* **490**, 49–54 (2012).

23. Almeida, D., Maldonado, E., Vasconcelos, V. & Antunes, A. Adaptation of the Mitochondrial Genome in Cephalopods: Enhancing Proton Translocation Channels and the Subunit Interactions. *PLOS ONE* **10**, e0135405 (2015).

24. Dierckxsens, N., Mardulyn, P. & Smits, G. NOVOPlasty: de novo assembly of organelle genomes from whole genome data. *Nucleic Acids Res.* **45**, e18–e18 (2017).

25. Grabherr, M. G. *et al.* Trinity: reconstructing a full-length transcriptome without a genome from RNA-Seq data. *Nat. Biotechnol.* **29**, 644–652 (2011).

26. Minh, B. Q. *et al.* IQ-TREE 2: New Models and Efficient Methods for Phylogenetic Inference in the Genomic Era. *Mol. Biol. Evol.* **37**, 1530–1534 (2020).

27. Kalyaanamoorthy, S., Minh, B. Q., Wong, T. K. F., Haeseler, A. von & Jermiin, L. S. ModelFinder: fast model selection for accurate phylogenetic estimates. *Nat. Methods* **14**, 587–589 (2017).

28. Hoang, D. T., Chernomor, O., von Haeseler, A., Minh, B. Q. & Vinh, L. S. UFBoot2: Improving the Ultrafast Bootstrap Approximation. *Mol. Biol. Evol.* **35**, 518–522 (2018).

29. Edgar, R. C. MUSCLE: multiple sequence alignment with high accuracy and high throughput. *Nucleic Acids Res.* **32**, 1792–1797 (2004).

30. Larsson, A. AliView: a fast and lightweight alignment viewer and editor for large datasets. *Bioinforma. Oxf. Engl.* **30**, 3276–3278 (2014).

31. Sanderson, M. J. r8s: inferring absolute rates of molecular evolution and divergence times in the absence of a molecular clock. *Bioinformatics* **19**, 301–302 (2003).

32. Tanner, A. R. *et al.* Molecular clocks indicate turnover and diversification of modern coleoid cephalopods during the Mesozoic Marine Revolution. *Proc. R. Soc. B Biol. Sci.* **284**, 20162818 (2017).

33. Anderson, F. E. & Marian, J. E. A. R. The grass squid Pickfordiateuthis pulchella is a paedomorphic loliginid. *Mol. Phylogenet. Evol.* **147**, 106801 (2020).

34. Shu, S., Goodstein, D. & Rokhsar, D. PERTRAN: Genome-guided RNA-seq Read Assembler. *Methods* (2013).

35. Haas, B. J. *et al.* Improving the Arabidopsis genome annotation using maximal transcript alignment assemblies. *Nucleic Acids Res.* **31**, 5654–5666 (2003).

36. Yeh, R. F., Lim, L. P. & Burge, C. B. Computational inference of homologous gene structures in the human genome. *Genome Res.* **11**, 803–816 (2001).

37. Salamov, A. A. & Solovyev, V. V. Ab initio Gene Finding in Drosophila Genomic DNA. *Genome Res.* **10**, 516–522 (2000).

38. Simão, F. A., Waterhouse, R. M., Ioannidis, P., Kriventseva, E. V. & Zdobnov, E. M. BUSCO: assessing genome assembly and annotation completeness with single-copy orthologs. *Bioinformatics* **31**, 3210–3212 (2015).

39. Sievers, F. *et al.* Fast, scalable generation of high-quality protein multiple sequence alignments using Clustal Omega. *Mol. Syst. Biol.* **7**, 539 (2011).

40. Price, M. N., Dehal, P. S. & Arkin, A. P. FastTree 2--approximately maximum-likelihood trees for large alignments. *PloS One* **5**, e9490 (2010).

41. Flynn, J. M. *et al.* RepeatModeler2 for automated genomic discovery of transposable element families. *Proc. Natl. Acad. Sci.* **117**, 9451–9457 (2020).

42. Smit, A., Hubley, R. & Green, P. Smit, AFA, Hubley, R & Green, P. RepeatMasker Open-4.0. *http://www.repeatmasker.org* (2013).

43. Yang, Z. PAML 4: Phylogenetic Analysis by Maximum Likelihood. *Mol. Biol. Evol.* **24**, 1586–1591 (2007).

44. Camacho, C. *et al.* BLAST+: architecture and applications. *BMC Bioinformatics* **10**, 421 (2009).

45. Simakov, O. *et al.* Deeply conserved synteny resolves early events in vertebrate evolution. *Nat. Ecol. Evol.* **4**, 820–830 (2020).

46. Simakov, O. *et al.* Insights into bilaterian evolution from three spiralian genomes. *Nature* **493**, 526–531 (2013).

47. Alon, S. *et al.* The majority of transcripts in the squid nervous system are extensively recoded by A-to-I RNA editing. *eLife* https://elifesciences.org/articles/05198 (2015) doi:10.7554/eLife.05198.
