# Supplementary material for: Genome and transcriptome mechanisms driving cephalopod evolution
Source: Nat Commun. 2022 May 4;13:2427. doi: 10.1038/s41467-022-29748-w (PMC9068888; doi:10.1038/s41467-022-29748-w)
Supplement: Supplementary file 2 — Reporting Summary [file 41467_2022_29748_MOESM2_ESM.pdf]

nature portfolio

Corresponding author(s): Clifton W Ragsdale, Oleg Simakov, Daniel S. Rokhsar

Last updated by author(s): Feb 14, 2022

Reporting Summary

Nature Portfolio wishes to improve the reproducibility of the work that we publish. This form provides structure for consistency and transparency in reporting. For further information on Nature Portfolio policies, see our Editorial Policies and the Editorial Policy Checklist.

Statistics

For all statistical analyses, confirm that the following items are present in the figure legend, table legend, main text, or Methods section.

n/a	Confirmed	
☐	☒	The exact sample size (*n*) for each experimental group/condition, given as a discrete number and unit of measurement
☐	☒	A statement on whether measurements were taken from distinct samples or whether the same sample was measured repeatedly
☐	☒	The statistical test(s) used AND whether they are one- or two-sided<br>*Only common tests should be described solely by name; describe more complex techniques in the Methods section.*
☒	☐	A description of all covariates tested
☒	☐	A description of any assumptions or corrections, such as tests of normality and adjustment for multiple comparisons
☐	☒	A full description of the statistical parameters including central tendency (e.g. means) or other basic estimates (e.g. regression coefficient) AND variation (e.g. standard deviation) or associated estimates of uncertainty (e.g. confidence intervals)
☐	☒	For null hypothesis testing, the test statistic (e.g. *F*, *t*, *r*) with confidence intervals, effect sizes, degrees of freedom and *P* value noted<br>*Give P values as exact values whenever suitable.*
☒	☐	For Bayesian analysis, information on the choice of priors and Markov chain Monte Carlo settings
☐	☒	For hierarchical and complex designs, identification of the appropriate level for tests and full reporting of outcomes
☐	☒	Estimates of effect sizes (e.g. Cohen's *d*, Pearson's *r*), indicating how they were calculated

Our web collection on statistics for biologists contains articles on many of the points above.

Software and code

Policy information about availability of computer code

Data collection	No software was used in data collection.
Data analysis	We used: hipmer(Georganas 2017), DBG2OLC(Ye 2016) (commit 1f7e752), platanus 1.2.4(Kajitani 2014), HiRise(Putnam 2014), juicebox toolkit(Durand 2016), wombat (https://gitlab.com/Bredeson/wombat), PBJelly(English 2012), minimap2(Li 2018), Quiver,(Chin 2013), freebayes(Garrison 2012) (v1.1.0-54-g49413aa), ILEC (v0.1.3; https://bitbucket.org/rokhsar-lab/map4cns), Lachesis (Burton et al. 2013), HiCPro (Servant 2015), NOVOplasty 4.0 (Dierckxsens et al. 2017), Trinity (Grabherr et al. 2011), ORFfinder (https://www.ncbi.nlm.nih.gov/orffinder/), NCBI BLAST v2.10 (https://ftp.ncbi.nlm.nih.gov/blast/executables/blast+/LATEST/), IQ-TREE v1.6.12 (Minh et al. 2020), ModelFinder (Kalyaanamoorthy et al. 2017), MUSCLE 3.8 (Edgar 2004), r8s 1.8 (Sanderson 2003), AliView (Larsson 2014), DOE Joint Genome Institute (JGI) annotation pipeline (img.jgi.doe.gov/docs/pipelineV5/), PERTRAN (Shu et al., 2013), PASA (Haas et al., 2003) GenomeScan (Yeh et al., 2001) Fgenesh+ (Salamov et al., 2000), BUSCO (Simao et al., 2015), CLUSTALO (Sievers et al., 2011), FastTree2 (Price et al., 2010), Figtree(http://tree.bio.ed.ac.uk/software/figtree/), RepeatModeler (2.0) (Smit et al. 2013-2015), RepeatMasker (open-4.0.7) (Smit and Hubley 2008-2015), NCBI/RMBLAST (2.6.0+), yn00 (PAML package V4.9e, Yang 2007), STAR aligner 2.5.3a (Dobin 2013), Picard (MarkDuplicates) (http://broadinstitute.github.io/picard/), mpileup (samtools v1.6, (Li 2009), SnpEff v.4.3 (Cingolani 2012), and bcftools (samtools v1.6, (Li 2009). Code used to compute visualize linkage group distribution, compute putative cephLGs and Dotplots/Fisher blobplots is available under https://bitbucket.org/viemet/public/src/master/CephChromosomes/. Code used in RNA editing analyses is available at https://github.com/sofiamedinaruiz/Squid_RNAediting and at https://bitbucket.org/viemet/public/src/master/CephChromosomes/ .

For manuscripts utilizing custom algorithms or software that are central to the research but not yet described in published literature, software must be made available to editors and reviewers. We strongly encourage code deposition in a community repository (e.g. GitHub). See the Nature Portfolio guidelines for submitting code & software for further information.

Data

Policy information about availability of data

All manuscripts must include a data availability statement. This statement should provide the following information, where applicable:

- Accession codes, unique identifiers, or web links for publicly available datasets
- A description of any restrictions on data availability
- For clinical datasets or third party data, please ensure that the statement adheres to our policy

The genome and transcriptome sequence reads generated in this study for D. pealeii are deposited as Bioproject PRJNA641326 (https://www.ncbi.nlm.nih.gov/bioproject/PRJNA641326). The genome assembly for O. bimaculoides generated in this study is deposited as Bioproject PRJNA808169 (http://www.ncbi.nlm.nih.gov/bioproject/808169). The E. scolopes sequence data used in this study is available under Bioproject PRJNA645380 (https://www.ncbi.nlm.nih.gov/bioproject/ PRJNA645380). The Octopus bimaculoides sequence data used in this study are available under Bioproject PRJNA270931 (https://www.ncbi.nlm.nih.gov/bioproject/270931). The D. pealeii sequenced data used in this study are available in the SRA under accession SRP044717. Source data are provided as a Source Data file.

Field-specific reporting

Please select the one below that is the best fit for your research. If you are not sure, read the appropriate sections before making your selection.

☐ Life sciences ☐ Behavioural & social sciences ☒ Ecological, evolutionary & environmental sciences

For a reference copy of the document with all sections, see nature.com/documents/nr-reporting-summary-flat.pdf

Ecological, evolutionary & environmental sciences study design

All studies must disclose on these points even when the disclosure is negative.

Study description	We sequenced and assembled the genomic DNA and RNA isolated from a single male Doryteuthis pealeii. We compared our genome assembly to other cephalopods.
Research sample	We isolated tissue for gDNA and RNA preparation from a single adult male Doryteuthis pealeii collected from the Vineyard Sound in October 2015. We also collected gDNA from a single adult male Doryteuthis opalescens collected from the Hopkins Marine Station in August 2016 and gDNA from an adult male Heterololigo bleekeri collected in Japan.
Sampling strategy	We isolated gDNA from a single individual to generate a high quality genome assembly by minimizing negative effects of heterozygosity and to be able to conduct RNA editing analysis.
Data collection	C. Albertin, J. Rosenthal, R. Hanlon, and S. Senft collected tissue and flash froze it on liquid nitrogen for nucleic acid isolation.
Timing and spatial scale	The single Doryteuthis pealeii was collected in October 2015.
Data exclusions	No data was excluded from the genome assembly. RNAseq reads from the blood, ink, and posterior salivary gland were excluded from the RNA editing analysis because the read depth was too low or it was isolated from a different individual.
Reproducibility	We verified the reproducibility of our RNA editing analysis by repeating our analyses with data deposited from Alon et al., 2015 (eLife).
Randomization	Our study employed single representative individuals and therefore did not require randomization.
Blinding	Blinding was not relevant to this study

Did the study involve field work? ☐ Yes ☒ No

Reporting for specific materials, systems and methods

We require information from authors about some types of materials, experimental systems and methods used in many studies. Here, indicate whether each material, system or method listed is relevant to your study. If you are not sure if a list item applies to your research, read the appropriate section before selecting a response.

Materials & experimental systems

n/a	Involved in the study
x	Antibodies
x	Eukaryotic cell lines
x	Palaeontology and archaeology
	x Animals and other organisms
x	Human research participants
x	Clinical data
x	Dual use research of concern

Methods

n/a	Involved in the study
x	ChIP-seq
x	Flow cytometry
x	MRI-based neuroimaging

Animals and other organisms

Policy information about studies involving animals; ARRIVE guidelines recommended for reporting animal research

Laboratory animals	This study did not involve laboratory animals
Wild animals	Genomic DNA for all shotgun sequencing was derived from a single male individual collected in October 2015 by otter trawl from Vineyard Sound, by the Marine Resources Center of the Marine Biological Laboratories, Woods Hole, MA. Adult Doryteuthis opalescens were caught by jig in Monterey Bay. Specimens were transported back to the lab and anesthetized with 2% ethanol in seawater and euthanized by decapitation and transecting the brain in accordance with guidelines on the ethical use of cephalopods.
Field-collected samples	Specimens were transported back to the lab and anesthetized with 2% ethanol in seawater and euthanized by decapitation and transecting the brain.
Ethics oversight	All work was performed in compliance with the EU Directive 2010/63/EU on cephalopod use and AAALAC guidelines on the care and welfare of cephalopods.

Note that full information on the approval of the study protocol must also be provided in the manuscript.
